# Identification of 4-phenylquinolin-2(1*H*)-one as a specific allosteric inhibitor of Akt

**DOI:** 10.1038/s41598-017-11870-1

**Published:** 2017-09-15

**Authors:** Bill X. Huang, Kenny Newcomer, Karl Kevala, Elena Barnaeva, Wei Zheng, Xin Hu, Samarjit Patnaik, Noel Southall, Juan Marugan, Marc Ferrer, Hee-Yong Kim

**Affiliations:** 1 Laboratory of Molecular Signaling, National Institute of Alcohol Abuse and Alcoholism, NIH, 5625 Fishers Lane, Rockville, MD 20852 USA; 2 Division of Preclinical Innovation, National Center for Advancing Translational Sciences, NIH, 9800 Medical Center Dr., Rockville, MD 20850 USA

## Abstract

Akt plays a major role in tumorigenesis and the development of specific Akt inhibitors as effective cancer therapeutics has been challenging. Here, we report the identification of a highly specific allosteric inhibitor of Akt through a FRET-based high-throughput screening, and characterization of its inhibitory mechanism. Out of 373,868 compounds screened, 4-phenylquinolin-2(1*H*)-one specifically decreased Akt phosphorylation at both T308 and S473, and inhibited Akt kinase activity (IC_50_ = 6 µM) and downstream signaling. 4-Phenylquinolin-2(1*H*)-one did not alter the activity of upstream kinases including PI3K, PDK1, and mTORC2 as well as closely related kinases that affect cell proliferation and survival such as SGK1, PKA, PKC, or ERK1/2. This compound inhibited the proliferation of cancer cells but displayed less toxicity compared to inhibitors of PI3K or mTOR. Kinase profiling efforts revealed that 4-phenylquinolin-2(1*H*)-one does not bind to the kinase active site of over 380 human kinases including Akt. However, 4-phenylquinolin-2(1*H*)-one interacted with the PH domain of Akt, apparently inducing a conformation that hinders S473 and T308 phosphorylation by mTORC2 and PDK1. In conclusion, we demonstrate that 4-phenylquinolin-2(1*H*)-one is an exquisitely selective Akt inhibitor with a distinctive molecular mechanism, and a promising lead compound for further optimization toward the development of novel cancer therapeutics.

## Introduction

Akt (protein kinase B) is a serine/threonine protein kinase that belongs to the AGC group within the eukaryotic protein kinase superfamily. Akt regulates various cellular processes including cell survival, growth, proliferation, migration, differentiation, and metabolism^1, 2^. It has been well established that hyperactivation of Akt is a major contributor to tumorigenesis and is associated with the resistance to chemotherapy and radiotherapy^3–6^. Thus, developing specific inhibitors of Akt is of great interest in cancer therapy^7^.

Akt consists of three conserved domains, an N-terminal pleckstrin homology (PH) domain, a central kinase domain which shares high homology with other AGC kinases, and a C-terminal regulatory domain containing a hydrophobic motif (HM)^8, 9^. The primary upstream activators of Akt are the phosphoinositide-3 kinases (PI3K) which generate phosphatidylinositol-3,4,5-trisphosphate (PIP_3_) upon growth factor stimulation^10^. The binding of PIP_3_ to the PH domain of Akt, together with the interaction of the membrane phosphatidylserine (PS) with both the PH and regulatory domains, recruits cytosolic Akt to the plasma membrane^11, 12^. The membrane-Akt interaction results in conformational changes of Akt^12, 13^, enabling its activation through phosphorylation at T308 in the kinase domain and at S473 of the C-terminal hydrophobic motif by 3-phosphoinositide dependent protein kinase-1 (PDK1) and mammalian target of rapamycin (mTOR)-rictor complex (mTORC2)^14, 15^, respectively.

Many Akt inhibitors under preclinical or clinical development are designed targeting the ATP binding pocket in the kinase domain^16^. These ATP-competitive inhibitors exhibit poor selectivity against closely related AGC kinases. Allosteric inhibitors of Akt with noticeably improved selectivity have also emerged^17–22^. By binding to the allosteric region located at the interface between the PH and kinase domains, this type of inhibitor prevents the membrane interaction or inter-domain conformational changes necessary for Akt activation^23–26^. Most inhibitors show adverse side effects such as hyperglycemia and toxicities exhibited at a similar extent to that of ATP-competitive inhibitors of PI3Ks^27^. Various clinical strategies have been applied to manage the toxicities of the inhibitors. For instance, intermittent dosing and standard medications such as loperamide are used to control rash and diarrhea respectively. Of note, phase I and II clinical studies with ATP-competitive inhibitors AZD5363 and ipatasertib have shown promising clinical activity in tumours with Akt1(E17K) mutations and in prostate cancers^28–32^. Allosteric inhibitors ARQ 092 and ARQ 751 also demonstrate promising clinical activity in patients with E17K Akt mutations and other pathway mutations^33^. Interestingly, ARQ 092 has also been used for the treatment of Proteus syndrome caused by Akt1 E17K mosaic activating mutation^34^. Nevertheless, no Akt inhibitor has gained clinical approval to date for the cancer treatment or other indication^27^.

Like many members of the AGC kinase family, the activation of Akt depends on the phosphorylation of the highly conserved T308 at the activation loop of the kinase domain. However, phosphorylation of S473 by mTORC2 has a distinctive influence on Akt activity^15^. It has been shown that S473 phosphorylation enhances Akt kinase activity by 4–5 fold by boosting the phosphorylation of T308^13, 15^. On the other hand, the inhibition of mTORC2 activity significantly reduces T308 phosphorylation by PDK1^35^. These findings indicate that S473 phosphorylation, as an important modulator of Akt activity, may be a potential therapetic target with fewer side effects.

In this report, we identified a highly specific Akt inhibitor by high throughput screening (HTS) based on such molecular basis. First, we developed a cell-based phospho-AKT (Ser473) assay, using the homogeneous time-resolved fluorescence (HTRF^®^) technology (Cisbio Bioassays,US) to detect S473 phosphorylation. Akt and S473-phosphorylated Akt were labeled with specific monoclonal antibodies that were conjugated with acceptor fluorophore d2 and donor europium^3+^ cryptate, respectively. When Akt is phosphorylated at S473, the time-resolved fluorescence resonance energy transfer (TR-FRET) from donor to acceptor occurs, generating the FRET signal. Through the HTS of a molecular library containing 373,868 compounds followed by validation with cell-based and biochemical assays and selectivity screening for the kinase activity of related kinases, we identified a specific non-ATP competitive inhibitor of S473 phosphorylation. We also unveiled a distinctive mechanism of action of the lead compound that may provide a molecular basis for further optimization of this novel class of specific Akt inhibitors aiming for more effective and less toxic cancer therapeutics.

## Results and Discussion

### Development of the HTS assay

**Figure 1:**
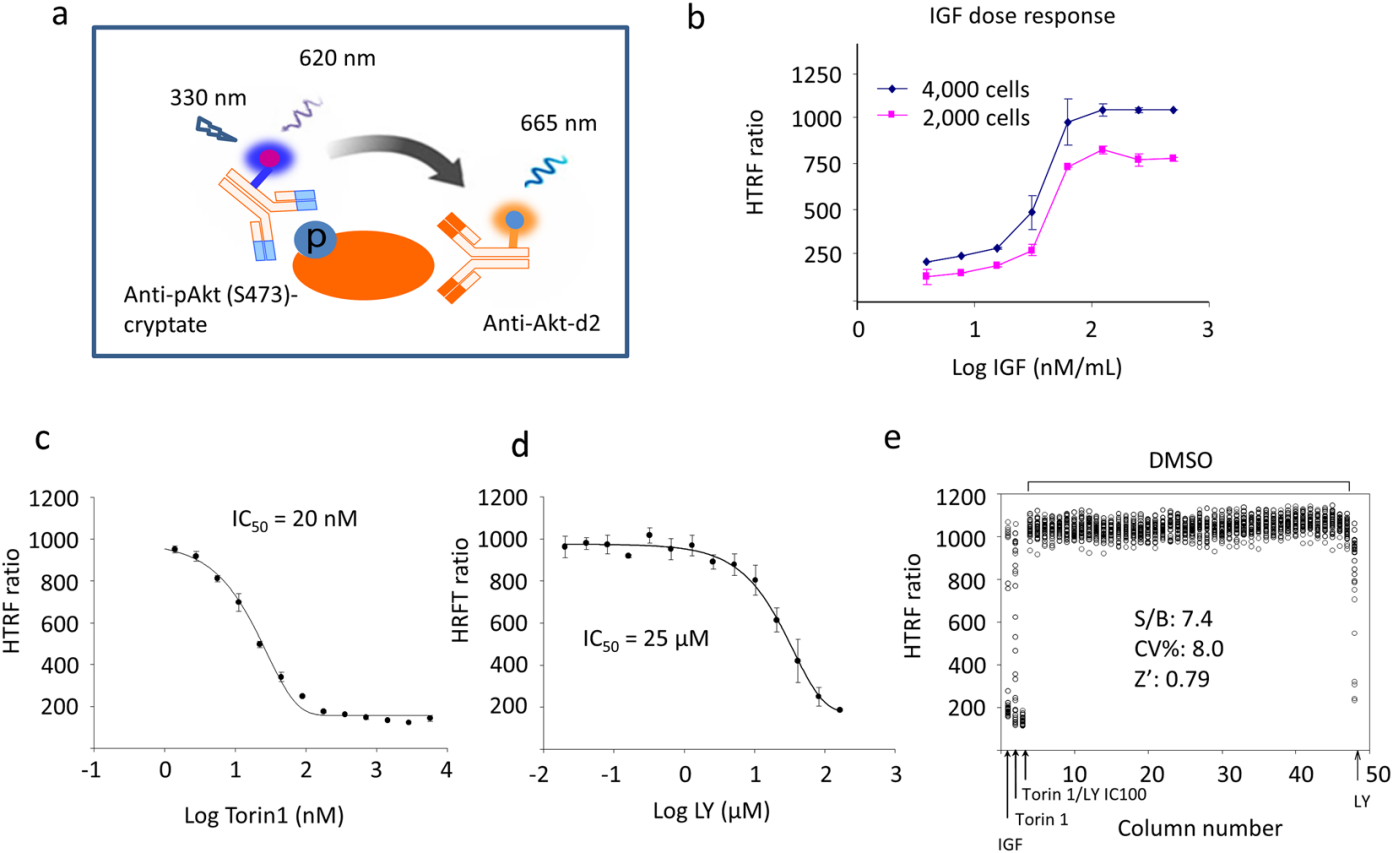
Development of the HTRF assay for Akt S473 phosphorylation. (**a**) Principle of the HTRF assay. An anti-pS473 antibody is labeled with a cryptate donor and anti-Akt antibody is labeled with acceptor d2. Upon excitation of donor at 330 nm, the donor generates long-lived emission at 620 nm. The energy is transferred from donor to acceptor when they are in close proximity (i.e., when they bind to the same Akt molecule), generating emission at 665 nm. The readout of HTRF ratio (665 nm/620 nm) indicates the extent of S473 phosphorylation. (**b**) Optimization of IGF stimulation for Akt phosphorylation at S473. (**c**,**d**) Validation of the HTS using known inhibitors. (**e**) Evaluation of the performance of the HTS assay. LY, PI3K inhibitor LY294002. IGF, insulin growth factor 1.

HTRF^®^ technology combines FRET between two nearby fluorophores with time-resolved fluorescence measurement that improves the sensitivity by eliminating the short-lived background fluorescence^36^. We devised an HTRF assay using fluorophore d2 conjugated to anti-Akt monoclonal antibody (Mab) and europium cryptate conjugated to anti-pS473-Akt Mab as the acceptor and donor fluorophores, respectively (Fig. 1a). To test the suitability of the HTRF^®^ assay, Neuro 2A cells were treated with insulin-like growth factor (IGF) which is known to phosphorylate Akt and the cell lysates were incubated with the fluorophore-conjugated antibodies. As expected, western blot analysis showed an elevated level of pS473 in response to IGF stimulation (Fig. S1a). The IGF-induced phosphorylation of S473 was detected by the HTRF^®^ assay as the readout fluorescence intensity ratio at 665/620 nm increased significantly compared to the unstimulated control (Fig. S1b). Of note, the HTRF ratio did not change significantly for more than 24 h. This stability made the scheduled reading for processing large numbers of HTS plates possible. To optimize the detection conditions, various cell densities, the time- and dose-response of IGF stimulation, as well as the cellular tolerance to DMSO, the compounds’ solvent, were tested first in a 384-well format (Fig. S2), and then adapted to the 1536-well format for increased high-throughput capacity. Briefly, the treatment of 4,000 cells per well in 1536-well plates with 200–500 ng/mL IGF for 40 min at 37 °C was found to generate the optimum HTRF signal (Fig. 1b). Up to 3% DMSO was found to have no impact on the assay. A final concentration of 0.57% DMSO was used for both control and the compound wells. The HTRF assay was further evaluated by using a known mTOR inhibitor, Torin 1^37^, and the commonly used PI3K inhibitor LY294002 (LY)^38^. In this case, Neuro 2A cells were preincubated with the inhibitors for 15 min at 37 °C prior to the stimulation with IGF. As expected, the HTRF ratio decreased with both inhibitors due to the inhibition of the upstream activators. The apparent IC_50_ values were determined to be ~20 nM (Fig. 1c, Fig. S2b) and 25 µM (Fig. 1d) for Torin 1 and LY respectively.

To evaluate the assay performance, a 1536-well DMSO test plate was tested (Fig. 1e). The Signal/Background ratio indicating the assay sensitivity and coefficient of variation (CV) were found to be 7.4 and 8.0% respectfully. The Z’ factor, an important assessment factor for both the dynamic range and variability of HTS assay, which should be ideally between 0.5–1.0, was calculated to be 0.79^39, 40^. These results demonstrated that the TR-FRET cell-based HTS assay for AKT pS473 is robust for 1563-well HTS. The assay was further validated by screening a small molecular library consisting of 1280 pharmacologically active compounds (LOPAC, Sigma-Aldrich) in a quantitative HTS format, where each compound was tested at seven concentrations, ranging from 4.90 nM to 76.6 µM (Fig. S3)^41^. A total number of three hits, or 0.24% of all compounds, were identified from this screening, which fell into the ideal hit rate of 0.1–0.5% for HTS. These data clearly indicated that the TR-FRET-based method developed is suitable for high throughput screening of the Akt inhibitors targeted at S473 phosphorylation.

### Primary HTS and secondary assays

HTS was performed against 373,868 compounds from the Molecular Libraries Small Molecule Repository (MLSMR) screened at a single dose of 50 µM. Nearly 3,000 primary hits were selected based on a cutoff greater than 30% assay inhibition. After filtering out false positive hits which demonstrated donor interference (i.e. affecting donor emission signal at 620 nm) and the compounds with reactive and promiscuous functional groups, a total of 1,858 inhibitory compounds were selected and re-tested for confirmation at 7 doses in the range between 76.6 µM and 4.90 nM in the same pAkt HTRF assay^41^. A total of 106 compounds were selected based on meeting all following criteria: 1) robust curve class categories (Curve Response Class or CRC: 1/2/3) and greater than 40% maximal response in confirmatory assay, 2) inactivity (CRC = 4) in both mock counter-screening and in cell viability assays. The inhibitory activity of these compounds was further evaluated using a cell-based ELISA assay and western blot analysis after which the number of compounds was further reduced to 75 for selectivity tests (Table S1).

### Tertiary screening for selectivity

**Figure 2:**
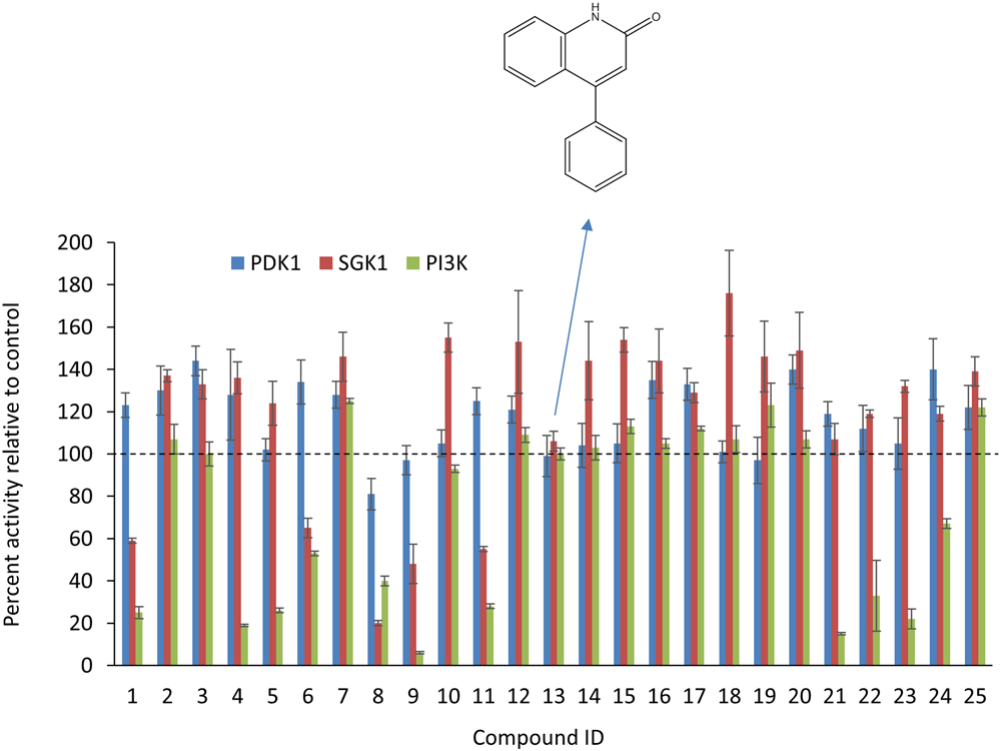
Screening for selective Akt inhibitors with no effects on PDK1, PI3K and SGK1 using ADP-Glo kinase assay. Shown are the results for representative compounds tested at 50 µM. Only compound #13, 4-phenylquinolin-2(1*H*)-one (G7), had no effects on any of these three kinases. PDK1, 3-phosphoinositide dependent protein kinase-1; PI3K, phosphoinositide-3 kinases; SGK1, serum/glucocorticoid regulated kinase 1. Inset, the chemical structure of G7.

A biochemical ADP-Glo kinase assay was performed for the 75 compounds to evaluate their effects on the enzymatic activity of recombinant PI3K and PDK1, upstream kinases of Akt, as well as serum/glucocorticoid regulated kinase 1 (SGK1) that shares the upstream kinases PDK1, and mTORC2 with Akt. The results from these kinase assays indicated that the compound #13, 4-phenylquinolin-2(1*H*)-one (G7), was the only inhibitor that affected none of these three kinases tested (Fig. 2). Based on the pS473-Akt HTRF assay, this compound has IC_50_ of 20 µM and maximum inhibition of 50% compared to that of 100 µM LY (Fig. S4).

### Inhibition of Akt phosphorylation evaluated by western blot analysis

**Figure 3:**
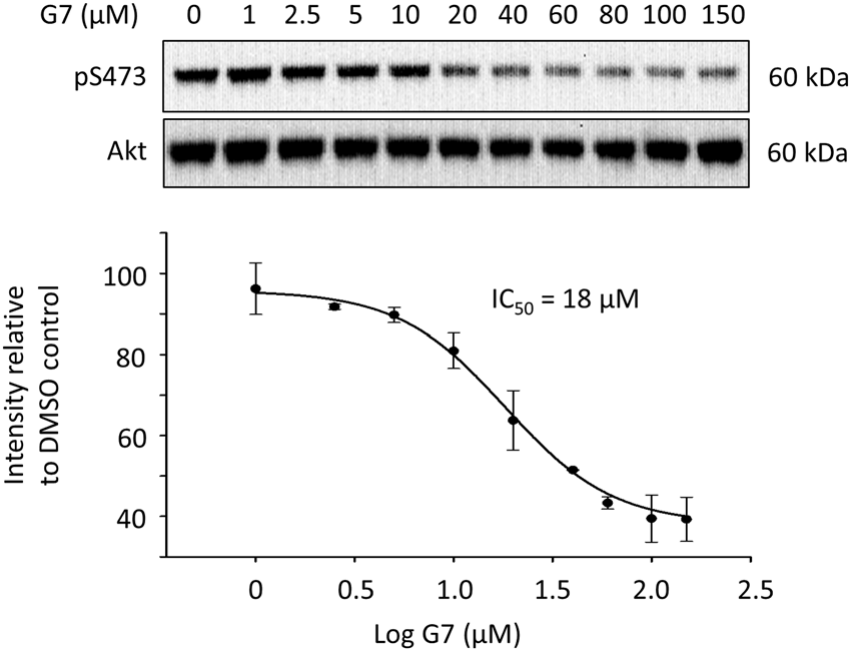
Evaluation of the inhibitory effect of G7 on cellular S473 phosphorylation. Neuro 2A cells were incubated with G7 at various concentrations for 30 min followed by IGF stimulation for 30 min. Cells were lysed and subjected to western blot analysis. Data represent means ± SEM of three independent experiments. G7, 4-phenylquinolin-2(1*H*)-one.

The inhibitory effect of G7 on Akt phosphorylation using the cell-based pS473-Akt HTRF assay was further evaluated by western blot analysis. Neuro 2A cells were treated with G7 at various concentration for 10 min before stimulation with IGF. As shown in Fig. 3, IGF-induced phosphorylation of S473 decreased with G7 in a dose-dependent manner. The IC_50_ was determined to be 18 µM which is in good agreement with the HTRF HTS result. Since S473 phosphorylation has been known to affect the phosphorylation of T308, we also examined the effect of G7 on T308 phosphorylation (Fig. S5). As expected, G7 decreased the T308 phosphorylation with an IC_50_ value similar to that observed for S473 phosphorylation. It is worth noting that G7 may also impair the phosphorylation of T308 directly.

### Inhibition of Akt kinase activity

**Figure 4:**
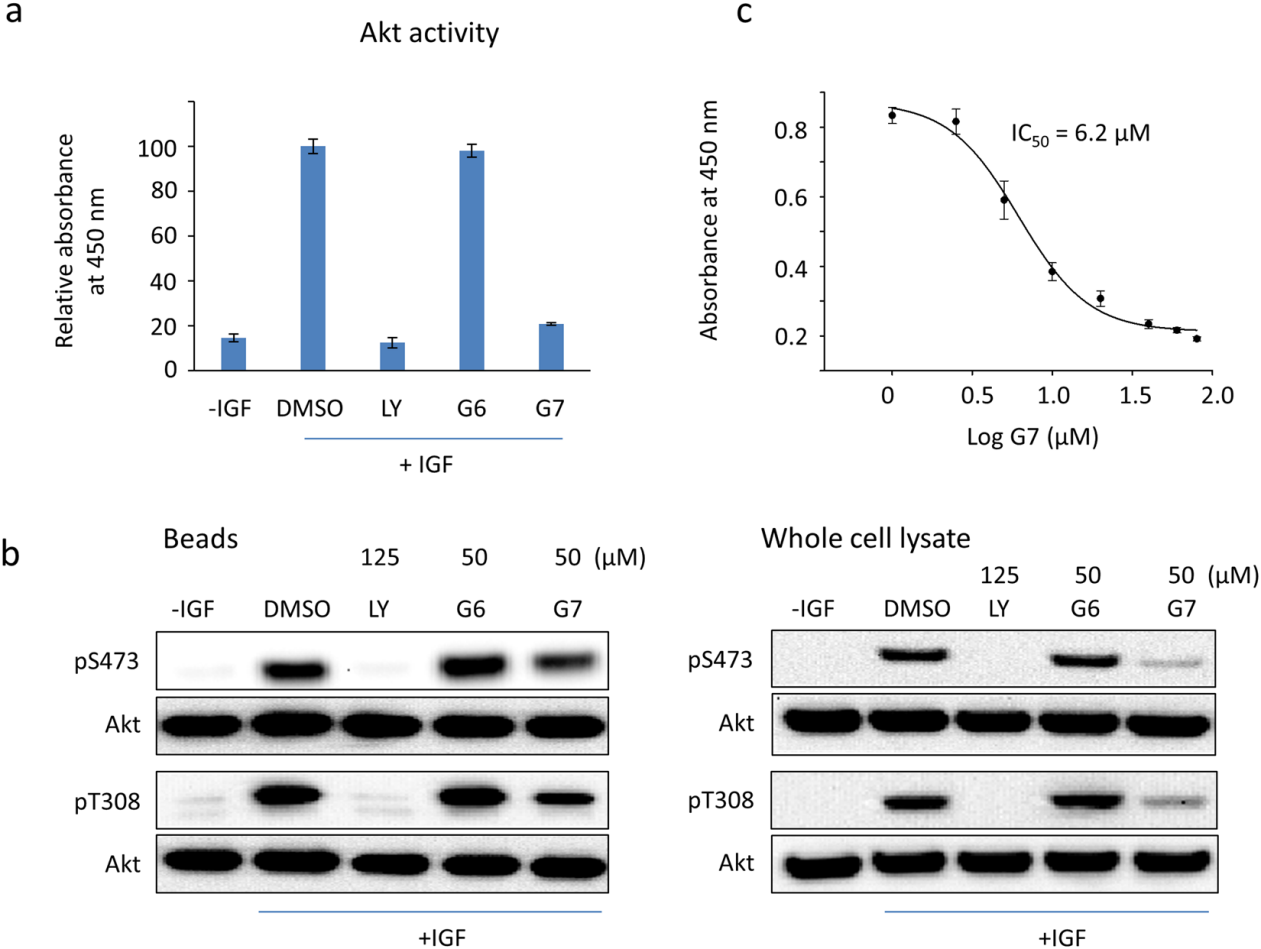
Inhibition of Akt activity by G7. Neuro 2A cells were incubated with inhibitors for 30 min prior to the stimulation with IGF (100 ng/mL). The activity of Akt immunoprecipitated on protein G beads was evaluated using K-Lisa Akt activity kit. (**a**) Akt activity affected by inhibitors. The Akt-specific activity was normalized to the level of immunopurified Akt on beads. (**b**) Western blot analysis of the immunopurified Akt. (**c**) Determination of IC_50_ of G7 in IGF-stimulated Neuro 2A cells based on Akt activity assay. LY, LY294002; G6, 1-(4-methylpiperazin-1-yl) propan-2-yl 2-chlorobenzoate; G7, 4-phenylquinolin-2(1*H*)-one.

To evaluate the inhibitory effect of G7 on the kinase activity of Akt, Akt was pulled down with an anti-Akt PH domain antibody from Neuro 2A cells that were pretreated with G7 at different concentrations and then stimulated with 100 ng/mL of IGF. The purpose of the immunoprecipitation step was to ensure not only the enrichment of the enzyme but also the measurement of Akt-specific activity. The capacity of the Akt immunopurified on protein G beads to phosphorylate a substrate peptide was evaluated by an ELISA-based assay. Figure 4a illustrates the results obtained from one of the G7 concentrations tested (50 µM). The amount of Akt immunoprecipitated was comparable for all samples tested, indicating that the epitope of the antibody did not interfere with the possible inhibitor-Akt interaction (Fig. 4b). Noticeably, G7-treated sample decreased significantly the IGF-induced phosphorylation at both S473 and T308, when compared to the sample without the inhibitor treatment (i.e., DMSO or G6, a control compound named 1-(4-methylpiperazin-1-yl)propan-2-yl 2-chlorobenzoate) (Fig. 4b). G7 at 50 µM decreased Akt activity by ~80%, while the positive control PIK3 inhibitor LY at 125 µM inhibited Akt activity by ~90%. With this approach, the IC_50_ of G7 on Akt enzymatic activity was determined to be 6 µM (Fig. 4c), which is similar to the IC_50_ of the PH-domain depedent Akt inhibitor Akt-I-1 and Akt-I-1,2 described previously^17^, or that of the widely used PI3K inhibitor LY294002^38^.

### Specific inhibitory effects of G7 on Akt signaling

**Figure 5:**
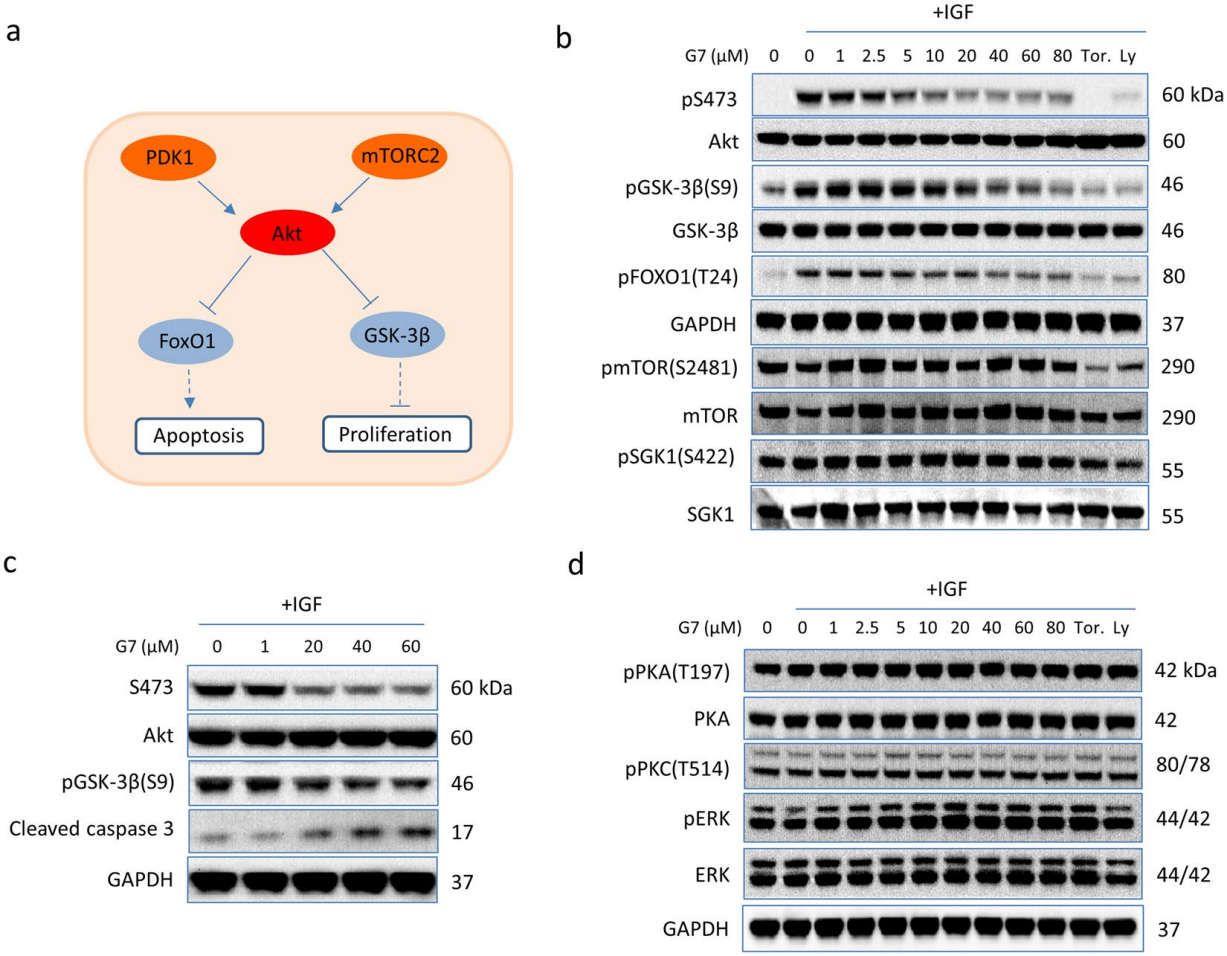
Effects of G7 on Akt-related signaling (**a**) Schematic presentation of Akt signaling depicting upstream activators and representative downstream effectors. (**b**,**c**) G7 inhibited Akt activation and Akt downstream signaling without affecting mTORC2 activation represented by S2481 phosphorylation of mTOR. (**d**) The inhibitor did not alter the phosphorylation thus the activation status of PKA, PKC and ERK1/2. G7, 4-phenylquinolin-2(1*H*)-one.

The primary function of Akt is to promote cell proliferation and cell survival by phosphorylating numerous downstream targets such as GSK-3β and the pro-apoptotic forkhead transcription factors (FoxO1/3a)^42, 43^(Fig. 5a). Indeed, phosphorylation of GSK-3β and FoxO1, decreased concurrently with S473 phosphorylation by G7 in a dose-dependent manner (Fig. 5b). Consequently, cells were more susceptible to apoptosis as indicated by the elevated levels of the cleaved caspase 3^12^ (Fig. 5c). However, G7 at all concentrations tested including 80 µM did not inhibit the phosphorylation of mTORC2 at S2481, nor the S422 phosphorylation of SGK1 which is another downstream substrate of mTORC2 (Fig. 5b), indicating that this inhibitor does not affect the activity of mTORC2. Moreover, G7 did not alter phosphorylation and thereby activation status of two closely related members of the AGC protein kinase family, namely PKC and PKA of which the catalytic domains are particularly similar to Akt. G7 neither inhibited the phosphorylation of ERK1/2 (Fig. 5d), another enzyme that influences cell proliferation and survival. All these data indicate that the newly identified inhibitor is highly specific to Akt activation and Akt-dependent cellular responses. Of note, the allosteric inhibitor MK-2206 has been shown to decrease mTORC2 activity despite high potency and high selectivity against 250 kinases^44^. To our knowledge, our data presents the first experimental demonstration for an Akt inhibitor that does not alter the activity of mTORC2.

### Interaction of G7 and Akt PH domain

**Figure 6:**
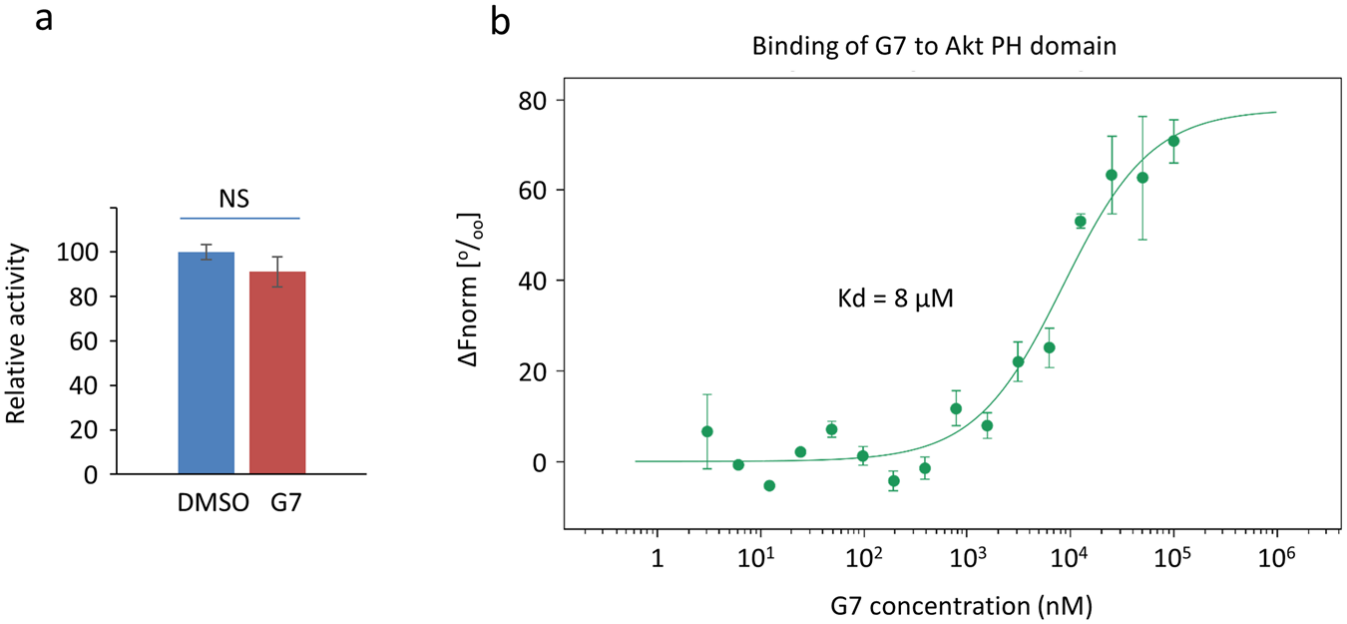
Interaction of G7 with Akt. (**a**) No effects of G7 at 20 µM on phosphorylation capability of activated Akt, indication that G7 do not compete with ATP or substrate binding to Akt. (**b**) Direct binding of G7 to the PH domain of Akt evaluated by microscale thermophoresis. Data are means ± SEM (n = 3). NS, not significant; G7, 4-phenylquinolin-2(1*H*)-one.

To understand the inhibitory mechanism of G7, possible direct interaction of this compound with Akt was tested by a biomolecular interaction analysis using microscale thermophoresis (MST)^45^. The changes in thermophoresis of the Akt molecules based on tryptophan- and tyrosine-derived fluorescence were measured to determine the binding constant^45^. The MST response curve shown in Fig. 6b indicated the binding of G7 to the PH domain of Akt with Kd of 8 µM.

The ineffectiveness of G7 on closely related kinases (e.g. PKA and PKC) suggests that G7 does not bind to the conserved ATP binding pocket of the kinases. In addition, the results of the KINOMEscan™ selectivity screen indicated that G7 did not bind significantly to the activated kinase domain of over 380 human kinases including Akt^46^ (Table S2). When activated Akt was incubated with ATP and a substrate peptide in the presence of 20 µM G7 and the phosphorylation of the substrate peptide was evaluated by an ELISA-based assay (Fig. 6a), only negligible change of the substrate phosphorylation was observed, indicating that G7 did not interfere with the ATP or substrate binding.

Although the interaction of G7 with the Akt regulatory domain has not been evaluated separately, the Kd values (4–6 µM) generated by the microscale thermophoresis for the G7 binding to full-length Akt (regardless of the activation status) were similar to that observed with the PH domain (Fig. S6), indicating that G7 mostly interacts with the PH domain of Akt.

### Effect of G7 on Akt phosphorylation by upstream kinases *in vitro*

**Figure 7:**
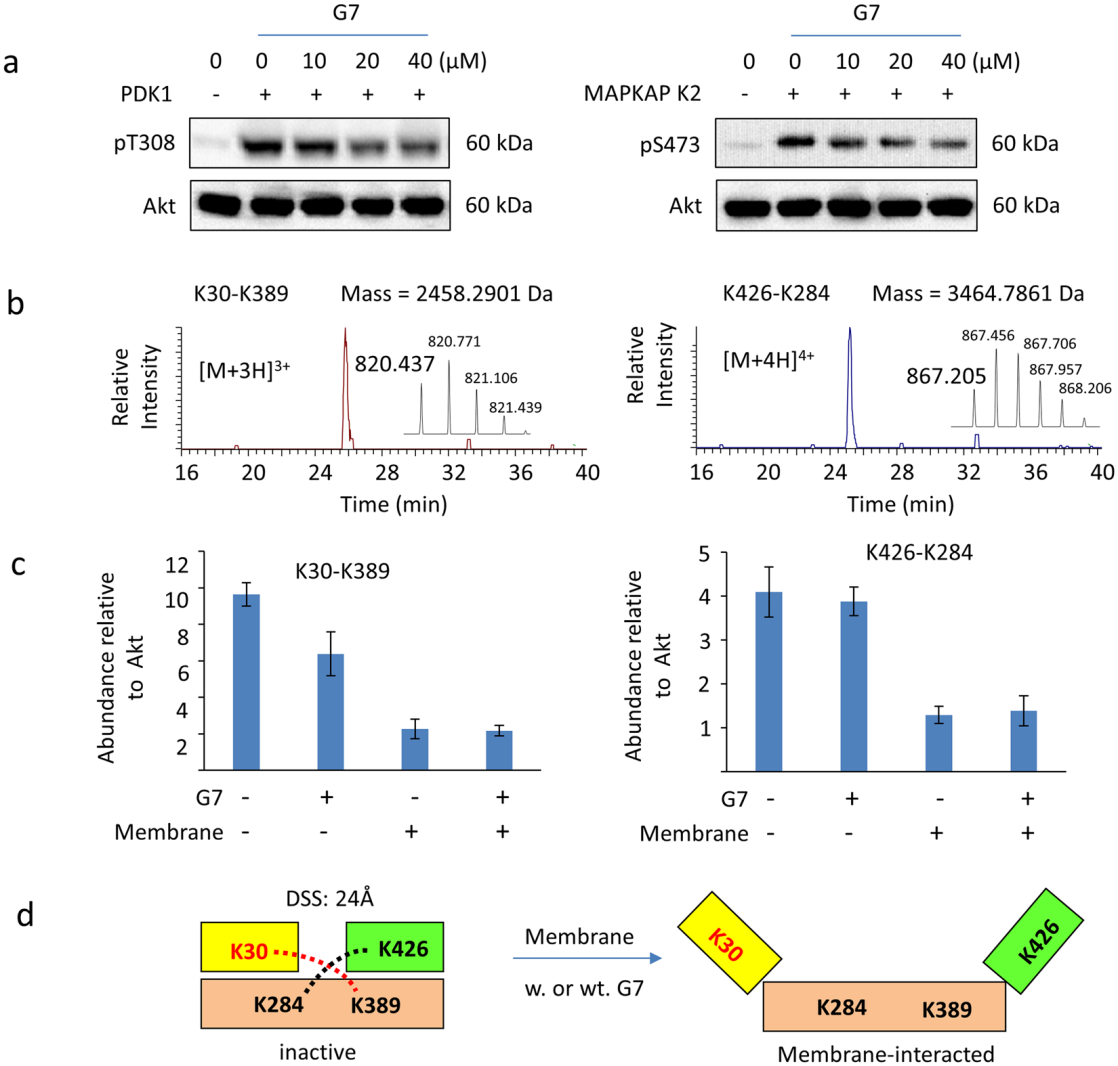
Effect G7 on interdomain conformational changes of Akt and its *in vitro* phosphorylation by upstream kinases. (**a**) G7 inhibits *in vitro* phosphorylation of T308 by PDK1 and S473 by MAPKAP kinase 2. (**b**) Mass spectrometric detection of two interdomain cross-linked peptides from inactive Akt. The peptide with mass of 2458.2901 Da reconstructed from triply charged ion at m/z 820.437 represents the cross-linking between K30 of peptide Y[26–39]K in the PH domain and K389 of peptide D[387–391] in the kinase domain (K30-K389). The peptide with mass of 3464.7861 reconstructed from quadruply charged ion at 867.205 represents the cross-linking between K426 of peptide L[421–436]R in the regulatory domain and K284 of peptide L[2777–289]K in the kinase domain (K426-K284). (**c**) Label-free quantitation of cross-linked peptides by Progenesis QI for Proteomics. (**d**) Schematic presentation of the interdomain conformations after Akt-membrane interaction. G7, 4-phenylquinolin-2(1*H*)-one; DSS, disuccinimidyl suberate.

As G7 is a PH domain-binding allosteric inhibitor of Akt, G7 is expected to result in conformational changes of Akt, which may in turn hinder its phosphorylation by upstream kinases PDK1 and mTORC2. To test this hypothesis, we examined *in vitro* phosphorylation of Akt by PDK1 or MAPKAP kinase 2 which can phosphorylate S473 *in vitro* ^12, 47^. Inactive Akt was incubated in the presence or absence of G7 with unilamellar vesicles containing PE/PC/PS/PIP_3_ (50%/19%/30%/1%), which mimicked a membrane composition in the inner leaflet of neuronal plasma membrane, followed by the addition of Mg^2+^/ATP and active PDK1 and MAPKAP kinase 2. As shown in Fig. 7a, phosphorylation of both T308 and S473 was impaired by the inhibitor. Considering that G7 does not affect the activity of PDK1 activity or MAPKAP kinase 2 (Fig. 2, Table S2), hindered accessibility of these upstream kinases to T308 and S473 of Akt was the only plausible explanation for the inhibited phosphorylation of Akt.

### Effect of G7 on the interdomain conformation of Akt

We next tested whether the inhibitor induced allosteric conformational changes that block the accessibility of the upstream kinases to T308 and S473. We used chemical cross-linking strategy combined with quantitative mass spectrometry that we have previously developed to discern detailed molecular mechanisms for the PH domain-binding inhibitors of Akt^48^. We quantitatively monitored the two interdomain cross-linked peptides, namely, K30 of the PH domain linked to K389 of the kinase domain (K30-K389), and K426 of the regulatory domain linked to K284 of the kinase domain (K426-K284), both via disuccinimidyl suberate (DSS), a lysine specific cross-linker enabling a maximum crosslinking distance of ~24 Å. The presence of K30-K389 and K426-K284 cross-linking observed in inactive (non-membrane-interacted) Akt molecules (Fig. 7b) indicated that the distance between the α-carbons of these cross-linked lysine pairs was within ~24 Å, revealing a folded structure where the PH domain and the kinase domain covered the part of the kinase domain^49, 50^. When Akt interacted with G7, the PH-kinase domain cross-linking (K30-K389) decreased, apparently due to the binding of the inhibitor to the PH domain (Fig. 7c). After Akt-membrane interaction, both interdomain cross-links decreased significantly as reported previously, suggesting that the PH and regulatory domains unfolded from the kinase domain^49, 50^. Of note, the open configuration exposes T308 and S473 for phosphorylation by PDK1 and mTORC2 respectively^12^. Despite the presence of G7, the decreased interdomain cross-linking induced by the membrane interaction remained unchanged. It is possible that the allosteric configurational changes induced by the inhibitor was too subtle to detect by the low-resolution cross-linking approach. Alternatively, G7 may not interfere with Akt-membrane interaction despite its binding to the PH domain. Indeed, G7 did not interfere with the PIP_3_ binding to Akt which is known to occur through the PIP_3_ binding pocket in the PH domain as evidenced by the insignificant effect of G7 on the Akt-PIP_3_ association revealed by a lipid-protein pull-down assay^51^ (Fig. S7). Likewise, the cross-linking data also suggested that G7 does not interfere with the key phosphatidylserine (PS)-binding residues such as K20 and R15 in the PH domain or K419/K420 in the regulatory domain which are also important for Akt phosphorylation and activation^12^.

We have previously demonstrated that the PH domain-dependent inhibitors can interfere with Akt-membrane interaction in different ways^48^. We showed that the binding of Akt to a phosphatidylinositol (PI) analog causes open interdomain conformations before interacting with membrane thus preventing the membrane translocation of Akt. Unlike the PI analog, a TCL1-peptide inhibitor does not alter the interdomain conformations before membrane interaction. Instead, the TCL1 peptide competes with the membrane interaction and consequently impairs the unfolding of the PH-kinase interdomain^48^. In addition, allosteric inhibitors including MK-2206 and Akt Inhibitor VIII (EMD Millipore) are thought to interact with both PH and the kinase domains promoting the formation of inactive conformation and impeding membrane translocation of Akt^23, 52^. Unlike these cases, G7 did not appear to affect the Akt-membrane interaction and the resulting interdomain conformational changes detected by the chemical cross-linking approach (Fig. 7c). Taken together, it is apparent that G7 causes distinct allosteric conformational changes that interferes with the accessibility of S473 and T308 to upstream enzymes for phosphorylation through the binding to the PH domain outside the PIP_3_ binding pocket.

### Effect of G7 on cell proliferation and cell viability

**Figure 8:**
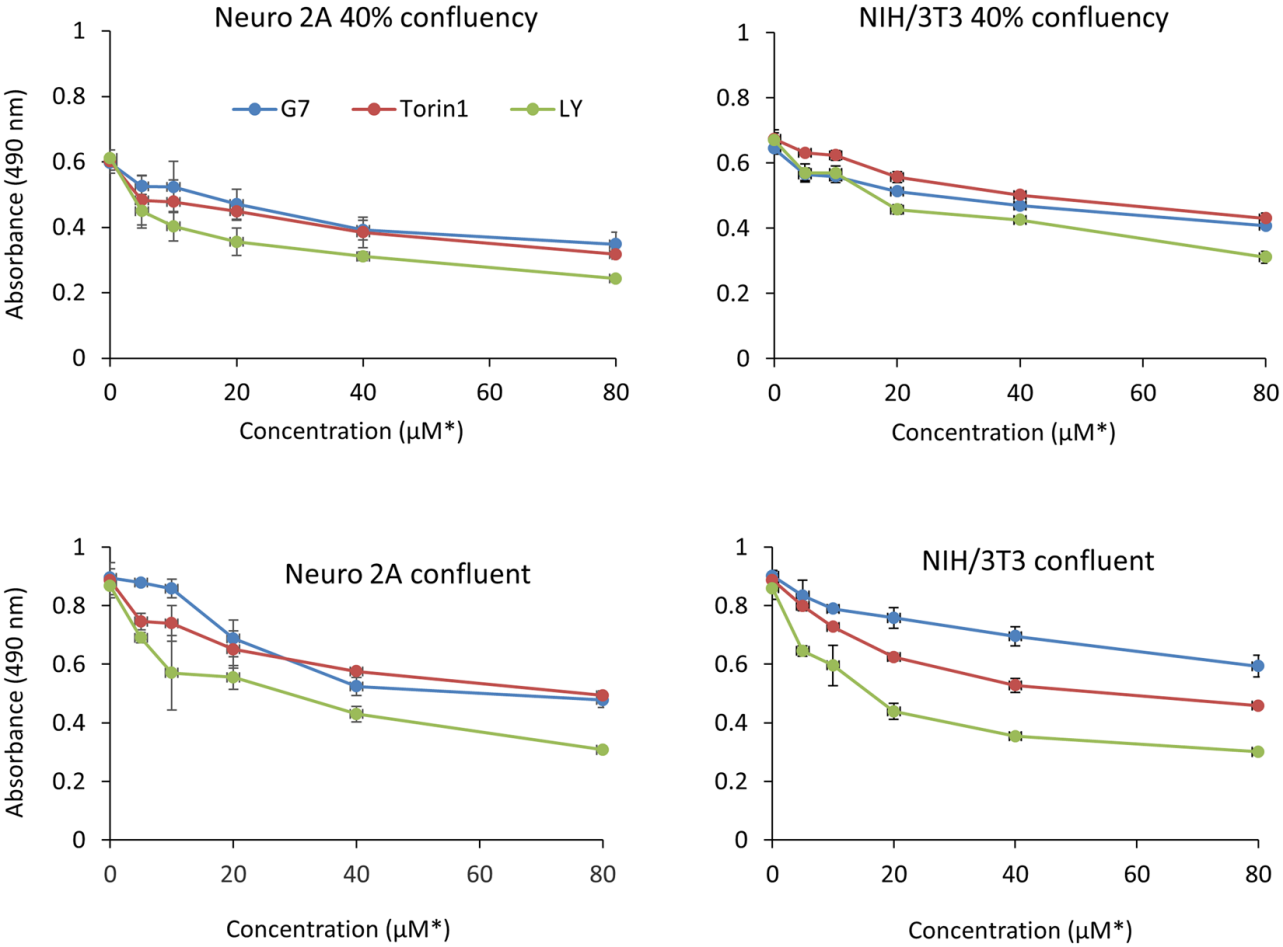
Effect of G7 on cell proliferation and viability. Cells seeded at different confluency were treated with inhibitors for 1 day and subjected to MTS assays. *µM for both G7 and LY and nM for Torin1. Data represent means ± SEM (n = 3). G7, 4-phenylquinolin-2(1*H*)-one, LY, LY294002.

The cellular effects of G7 were examined for the proliferation of Neuro 2A neuroblastoma cancer cells and viability of non-cancerous NIH/3T3 cells using the MTS assay. NIH/3T3 fibroblast cells are highly contact-inhibited and stop proliferating when they reach full confluency. In contrast, cancer cells like Neuro 2A have lost the contact-inhibition property, and thus continue to proliferate with no control. G7 inhibited the proliferation of Neuro 2A cells (Fig. 8) and LNCaP prostate cancer cells (Fig. S8), as well as the ATP-competitive PI3K inhibitor LY or mTOCR2 inhibitor Torin1. When NIH/3T3 cells were at a proliferation stage (e.g. at 40% confluency), all three inhibitors tested showed similar inhibitory effect. However, when the 3T3 cells were confluent and no longer proliferated, considerably less inhibition was observed with G7 in comparison to LY or Torin 1. These data suggest that G7 is less cytotoxic to non-cancerous cells while maintaining the anti-cancer properties of an Akt inhibitor.

In conclusion, we have identified an allosteric Akt inhibitor with excellent selectivity and less toxicity, presenting a promising lead compound for further optimization and development of novel therapeutic agents against cancers.

## Methods

### Cell culture and IGF stimulation

Mouse neuroblastoma Neuro 2A cells (ATCC) were maintained in Dulbecco’s Modified Eagle Medium (DMEM) containing 5% fetal bovine serum (FBS) in a humidified atmosphere supplied with 5% CO_2_ at 37 °C. Cells were starved overnight in serum-free DMEM before treatment with or without inhibitors followed by stimulation with recombinant human insulin-like growth factor-1 (IGF-1, PeproTech).

### HTRF assay

Neuro 2A cells were seeded (4,000 cells/well) in 4 µL of DMEM with 0.5% FBS and 1x Penicillin/Streptomycin in white solid bottom tissue-culture treated 1536-well plates (Greiner, 789173-F) with MultiDrop Combi Dispenser (Thermo Scientific). The cells were incubated for 24 h at 37 °C, 5% CO_2_, 95% humidity. Twenty-three nL of tested compounds were transferred to each well with an automated pin-tool station (Kalypsys). Following incubation for 15 min at 37 °C, 0.5 µL IGF (500 ng/mL of final concentration) was added and incubated for additional 40 min at 37 °C. After the addition of 1.5 µL 4x lysis buffer and 15-min incubation at room temperature (25 °C), 2 µL of HTRF^®^ conjugated antibodies (fluorophore d2 conjugated anti-Akt monoclonal antibody and Europium^3+^ cryptate-labeled anti-pS473-Akt monoclonal antibody, obtained from CisBio, 1:80 dilution) were added with BioRAPTR FRD™ (Beckman Coulter). The plates were incubated at room temperature for 20 h. The signal was measured by EnVision Multilabel Plate Reader (PerkinElmer) with excitation at 330 nm, emission at 620 nm (donor) and 665 nm (acceptor). Results were calculated and analyzed in a format of the ratio 665 nm/620 nm multiplied by 10^4^.

Relative activity was calculated by normalizing each HTRF signal from each sample well to the mean HTRF signal from the DMSO-only control wells.

### Akt activity assay

After overnight starvation by incubating with serum-free DMEM, Neuro 2A cells were treated with compound G7 at various concentrations for 30 min at room temperature followed by stimulation with IGF (final concentration of 100 ng/mL) for 30 min. Cells were lysed with lysis buffer containing 1% Triton X-100 and protease/phosphatase inhibitors (Cell Signaling Technology) and subjected to immunoprecipitation using protein G-plus and anti-AKT PH domain (Millipore). The activity of the immunopurified Akt was evaluated using a K-Lisa Akt activity kit (Millipore) in accordance with manufacturer’s instructions. The activity was normalized to Akt level in the immunoprecipitates determined by quantitative western blot analysis.

### Microscale thermophoresis (MST)

Using 0.1 M Tris-HCl buffer (pH 7.2) containing 0.05% Tween-20, a 1:1 serial dilution was prepared for G7 to yield 16 working solutions, with the highest concentration being 200 µM. Each of these G7 solutions was mixed with 1 µM of purified Akt sample prepared in the same 0.05% Tween-20 Tris-HCl buffer. The Akt samples tested include recombinant Akt PH domain, inactive Akt, and active Akt obtained from Millipore. After 15-min incubation the samples were centrifuged at 14,000 g at 4 °C before loading into premium coated capillaries (NanoTemper Tech.) for MST measurements. The MST experiments was conducted on a Monolith NT.LabelFree instrument (NanoTemper Tech.), at 20% LED power and 40% MST power. Data analyses were performed using the NanoTemper analysis software.

### MTS cell proliferation/viability assay

Neuro 2A cells or NIH/3T3 cells (ATCC) were seeded at various confluency in a 96-well plate in 100 µL DMEM containing 5% FBS or 10% FBS with 1% penicillin streptomucin, respectively, at 37 °C in a 5% CO_2_ and 95% humidity incubator for 18 hr. To each well 1 µL inhibitor with various concentrations or DMSO was added. The final concentrations of the tested inhibitors were 0, 5, 10, 20, 40 and 80 µM for G7 and LY, and 0, 5, 10, 20, 40 and 80 nM for Torin1, based on their respective IC_50_ values (~µM for G7 and LY and ~ nM for Torin 1). After 24 hr, 20 µL of CellTiter 96® AQueous One Solution Reagent (Promega) were added. The plate was placed in the incubator for 2 h and absorbance at 490 nm was measured with a Synergy HT Microplate Reader (Bio-Tek).

The following methods are included in Supporting information: Compound library, Quantitative HTS (qHTS) and curve response class classification, Hit selection criteria, Western blot analysis, Elisa Assay for phosphorylation of Akt, ADP-Glo kinase assay, KINOMEscan™ selectivity screen, Preparation of unilamellar vesicles, Akt-membrane interaction and *in vitro* phosphorylation, PIP_3_-Akt pull-down assay, Chemical cross-linking and mass spectrometry, and Supplemental figures including all uncropped western blots.

### Data availability statement

The data that support the findings of this study are available in Supplementary Information.

## Electronic supplementary material

Supporting information
