# Supplementary material for: Identification of 4-phenylquinolin-2(1H)-one as a specific allosteric inhibitor of Akt
Source: Sci Rep. 2017 Sep 15;7:11673. doi: 10.1038/s41598-017-11870-1 (PMC5601486; doi:10.1038/s41598-017-11870-1)

Supporting Information

Identification of 4-phenylquinolin-2(1H)-one as a specific allosteric inhibitor of Akt

Bill X. Huang[1], Kenny Newcomer[1], Karl Kevala[1], Elena Barnaeva[2], Wei Zheng[2], Xin Hu[2], Patnaik, Samarjit[2], Noel Southall[2], Juan Marugan[2], Marc Ferrer[2], Hee-Yong Kim[1]

[1]Laboratory of Molecular Signaling, National Institute of Alcohol Abuse and Alcoholism, NIH, Bethesda, MD 20892-9410
[2]Division of Preclinical Innovation, National Center for Advancing Translational Sciences (NCATS), NIH, 9800 Medical Center Dr, Rockville, Maryland 20892, USA.

***Running title**:*

***Key words**:*

Correspondence should be addressed to:

Hee-Yong Kim, Ph.D.

Laboratory of Molecular Signaling

National Institute of Alcohol Abuse and Alcoholism

National Institutes of Health

5625 Fishers Lane, Rm. 3N-07

Bethesda, MD 20892-9410

301-402-8746 (Tel)

301-594-0035 (Fax)

hykim@nih.gov

Methods

Compound library

The Library of Pharmacologically Active Compounds (LOPAC, 1280 compounds) and the Molecular Library Small Molecular Repository Library (MLSMR, 373,868 compounds) were obtained from Sigma-Aldrich.

Quantitative HTS (qHTS) and curve response class classification

The qHTS screen was performed as described previously[1]. Each compound was titrated in seven concentrations. The final concentrations of the compounds assayed ranged from 4.9 nM to 76.6 μM. The curve response classifications (CRC), which can be used to depict potency, efficacy and reliability of the data, and to estimate an IC_{50} value, were determined by a customized software[2]. The maximum response (100% inhibition) was set to the response to 50 μM LY-294002 (LY, purchased from Sigma-Aldrich) and the basal response (0% inhibition) was set to the DMSO control. Efficacy of a compound refers to the highest response normalized to the maximal response of LY. The CRCs were grouped into 4 categories. Class 1 are well-fit and have both upper and lower asymptotes with efficacy greater than 30% (Class1.1, >80% efficacy; Class 1.2; 30-80% efficacy). Class 2 are incomplete with only one asymptote (Class 2.1, > 80% efficacy; Class 2.2, 30-80% % efficacy). Class 3 display activity only at the highest concentrations and efficacy >30%. Class 4 are with low efficacy (<30%) or no response and considered inactive[3].

Hit selection criteria

The following criteria were applied for hit selection from the primary screen: 1, hits in robust curve classes (1/2/3) with efficacy greater than 30% were considered active; 2, these hits were filtered for donor interference; 3, hits were further filtered by reactive and promiscuous functional groups, as previously described[4]; 4, hits were clustered by structural similarity using Leadscope Hosted Client (Leadscope Inc., Columbus, OH)[2].

Western blot analysis

Neuro 2A cells were seeded in 12-well plates in DMEM containing 5% FBS for 24 hr. After overnight serum starvation (in 0% FBS DMEM), cells were incubated for 30 min at 37 °C with G7 or LY294002 or Torin 1 (Sigma Aldrich) at various concentrations, or DMSO control. IGF-1 was added to each well at a final concentration of 100 ng/mL. After 30-min incubation cells were washed with cold PBS and lysed with 50 μL lysis buffer containing 1% Triton X-100 and protease/phosphatase inhibitors (Cell Signaling Technology) or RIPA buffer (Santa Cruz Biotech). Samples were electrophoresed in 4–12% Bis-Tris gels (Life Technologies) at 150 V using MOPS SDS running buffer. Proteins were transferred electrophoretically to a PVDF or nitrocellulose membrane. For detection of cleaved

caspase 3 a Trans-Blot Turbo Transfer System (Bio-Rad, 25 V for 25 min) with PVDF membrane were used. Alternatively, for the detection of mTOR and phospho-mTOR (Ser2481), proteins extracted with RIPA buffer were transferred to a nitrocellulose membrane with NuPAGE transfer buffer (Life Technologies) for 60 min at 30 V followed by additional 60 min at 60 V. The membrane was blocked with 5% milk in TBS containing 0.1% Tween 20 (TBS-T) at room temperature for 1 hr. Blots were incubated with anti-Akt pS473 (#9271), anti-Akt pT308 (#2965), anti-mTOR pS2481 (#2974), anti-SGK pS422 (Abbexa, #012084), anti-FOXO1(pT24)/FOXO3(pT32) (#9464), anti-PKA pT197 (#5661), anti-PKC(pan) pT514 (# 9379), anti-GSK-3β pS9 (#9336), anti-p44/42 MAPK (Erk1/2) pT202/T204 (#4377), anti-Akt antibody (#9272), anti-mTOR (#2972), anti-SGK1 (Abbexa, #000966), anti-PKA C-α (#4782), anti-p44/42 MAPK (Erk1/2) (#4695), anti-GSK-3β (BD Biosciences, # 610201) or anti- GAPDH (14C10) antibody (#2118) (all antibodies were from Cell Signaling Technology unless indicated, 1:1000 dilution was used) at 4°C overnight, washed three times with TBS-T, then incubated with peroxidase-conjugated secondary antibody (Sigma-Aldrich) for 1 hr at room temperature. After washing with TBS-T, blots were incubated with ECL detection reagent (Thermo Scientific) for 5 min, and imaged with a Kodak Gel Logic 440 Imaging. The Restore Plus western blot stripping buffer (Thermo Scientific, #46430) was used in re-probing the blots, typically for re-probing the expression level of individual proteins after the detection of their phosphorylated form. Band intensity was quantitated using Kodak 1D imaging analysis software. All uncropped western blots can be found in Fig. S9.

Elisa Assay for phosphorylation of Akt

Neuro 2A cells in 48-well plates were incubated with the inhibitor for 30 minutes at 37°C followed by IGF stimulation for 30 min. Cells were lysed and subjected to Elisa assay using an Akt (S473) InstantOne Elisa kit (ThermoFisher Scientific, # 8586042-11) in accordance with manufacturer's instruction.

ADP-Glo kinase assay

An ADP-GloTM kinase assay kit (Promega, # V9101) was used to measure the activity of PDK1 or SGK1 in accordance with the manufacturer's instructions. For PDK1 assay, the reaction mixture included 5 μM ATP, 50 μM inhibitor, 100 ng recombinant PDK1 and 2 μg PDKtide substrate (Promega, #V2761) prior to the addition of the ADP-Glo reagents. For SGK1, 5 μM ATP, 50 μM inhibitor, 10 ng SGK1 and 1 μg Akt substrate peptide (Promega, #V2911) were used. Staurosporine at concentration between 0.4 and 4,000 nM or the SGK1 inhibitor GSK-650394 (Tocris Bioscience) between 7 and 2,000 nM were used as controls for PDK1 or SGK1 assay respectively. For PI3K assay, 50 μM ATP, 50 μM inhibitor, 100 ng recombinant PI3 kinase (SignalChem, Richmond, Canada, # P27-10H-05) and 1.5 mg/mL multilamellar phospholipid vesicles containing PC/PE/PS/PIP2 (at mass ratio of 1/0.5/0.2/0.1) were used. Inhibitor blanks as well as LY-294002 between 78 and

125,000 nM were used as controls.

KINOMEscan™ selectivity screen

The assay was performed at DiscoveRx Corporation according the methods published previously[5]. Briefly, Kinase-tagged T7 phage strains were grown in parallel in an *E. coli* host derived from the BL21 strain. *E. coli* were grown to log-phase and infected with T7 phage from a frozen stock and incubated with shaking at 32°C until lysis (90-150 minutes). The kinases were produced in HEK-293 cells and subsequently tagged with DNA for qPCR detection. Streptavidin-coated magnetic beads were treated with biotinylated small molecule ligands to generate affinity resins for kinase assays. The binding of kinases to liganded-affinity beads in the presence of 50 μM inhibitor or DMSO control was performed in polypropylene 384-well plates for 1 hr at room temperature. After wash with PBS buffer containing 0.05% Tween 20, the beads were incubated in elution buffer (PBS containing 0.05 % Tween 20 and 0.5 μM non-biotinylated affinity ligand) for 30 minutes at room temperature. The kinase concentration in the eluates was measured by qPCR.

Preparation of unilamellar vesicles, Akt-membrane interaction and in vitro phosphorylation

Unilamellar vesicles containing PE (18:0, 22:6)/PC (16:0, 18:1)/PS (18:0, 22:6)/PIP3 (18:0, 20:4) at a ratio of 50%/19%/30%/1%, which approximates a membrane composition in the inner leaflet of neuronal plasma membrane, were prepared

according to the method reported previously[6]. Briefly, the lipids dissolved in methanol/chloroform were mixed at desired proportions and dried with a N_2 steam. The lipid mixture was redissolved in 2 mL cyclohexane containing 75 μM BHT (2,6-di-tert-butyl-p-cresol) and lyophilized under vacuum. Unilamellar vesicles were obtained by reconstituting the dried sample with 1 mL of PBS (pH 7.4) containing 50 μM DTPA (diethylenetriamine pentaacetic acid) followed by extrusion with 0.1 μm polycarbonate membrane (Corning, Inc.). For *in vitro* phosphorylation of Akt, 1 μL of inactive Akt (Millipore, #14-279) at 0.1 μg/ μL was incubated with 20 μL of liposomes (unilamellar vesicles), 0.2 μL of inhibitor with varied concentrations, 3 μL of Mg^{2+}/ATP cocktail (Millipore, # 20-113), and 5 μL of 10 ng/ μL PDK1 (Millipore, # 14-452) or MAKAP kinase 2 (Millipore, # 14-337) for 40 min at 30 ^{o}C. The mixture was resolved on SDS gel followed by western blot analysis.

PIP3-Akt pull-down assay

The assay was performed using a protocol published previously[7]. Briefly, inactive Akt (50 ng) in PBS containing 400 ng/mL BSA was incubated with inhibitor G7 or a PI analog (EMD Millipore, #124005) for 2 hr at 4 ^{o}C. 25 μL PIP3 beads (Echelon, #P-B345a) was added to the sample and incubated overnight at 4 ^{o}C. The beads were washed with washing buffer containing 10 mM HEPES (pH 7.5), 0.25% NP-40 and 150 mM NaCl. Akt was eluted with 2x LDS and measured by immunoblotting.

Chemical cross-linking and mass spectrometry

Five μL of Akt (at 2.5 μM) was incubated with 30 μL unilamellar vesicles liposomes in the presence or absence of 20 μM inhibitor (G7) at 30 ^{o}C for 40 min. Alternatively, Akt was incubated with the inhibitor in 30 μL PBS buffer. The sample was incubated with a 50-molar excess of freshly prepared disuccinimidyl suberate (DSS) (ThermoFisher Scientific) in DMSO (a final concentration of DMSO of 3%) at room temperature for 30 min. The cross-linking reaction was quenched by adding 1 M Tris-HCl (pH 7.4) to a final concentration of 50 mM. The protein was digested with sequence grade trypsin (Promega). After desalting with a C18 Ziptip pipette tip (Millipore), the peptides were subjected to Nano-LC-ESI-MS/MS analysis performed on an LTQ-Orbitrap XL mass spectrometer (Thermo Scientific) equipped with an Eksigent nanoLC 1D system. The mobile phases consisted of 0.1% formic acid (solvent A) and 0.1% formic acid in 95% ACN (solvent B). Peptides were loaded onto a C18 trap column (Eksigent) and separated by a 15 cm IntegraFrit column (ProteoPep 2, New Objective) at a flow rate of 300 nL/min with a gradient from 3-50% solvent B in 50 min. Cross-linked peptides were quantified by Progenesis QI Proteomics. Two independent experiments were performed.

References

1. Inglese, J.; Auld, D. S.; Jadhav, A.; Johnson, R. L.; Simeonov, A.; Yasgar, A.; Zheng, W.; Austin, C. P., Quantitative high-throughput screening: A titration-based approach that efficiently identifies biological activities in large chemical libraries. *Proceedings of the National Academy of Sciences of the*

United States of America **2006,** *103* (31), 11473-11478.
2. Bhattacharyya, N.; Hu, X.; Chen, C. Z.; Griner, L. A. M.; Zheng, W.; Inglese, J.; Austin, C. P.; Marugan, J. J.; Southall, N.; Neumann, S.; Northup, J. K.; Ferrer, M.; Collins, M. T., A High Throughput Screening Assay System for the Identification of Small Molecule Inhibitors of gsp. *PloS one* **2014,** *9* (3).
3. Hu, Z. Y.; Hu, X.; He, S. S.; Yim, H. J.; Xiao, J. B.; Swaroop, M.; Tanega, C.; Zhang, Y. Q.; Yi, G. H.; Kao, C. C.; Marugan, J.; Ferrer, M.; Zheng, W.; Southall, N.; Liang, T. J., Identification of novel anti-hepatitis C virus agents by a quantitative high throughput screen in a cell-based infection assay. *Antivir Res* **2015,** *124*, 20-29.
4. Baell, J. B.; Holloway, G. A., New Substructure Filters for Removal of Pan Assay Interference Compounds (PAINS) from Screening Libraries and for Their Exclusion in Bioassays. *J Med Chem* **2010,** *53* (7), 2719-2740.
5. Fabian, M. A.; Biggs, W. H.; Treiber, D. K.; Atteridge, C. E.; Azimioara, M. D.; Benedetti, M. G.; Carter, T. A.; Ciceri, P.; Edeen, P. T.; Floyd, M.; Ford, J. M.; Galvin, M.; Gerlach, J. L.; Grotzfeld, R. M.; Herrgard, S.; Insko, D. E.; Insko, M. A.; Lai, A. G.; Lelias, J. M.; Mehta, S. A.; Milanov, Z. V.; Velasco, A. M.; Wodicka, L. M.; Patel, H. K.; Zarrinkar, P. P.; Lockhart, D. J., A small molecule-kinase interaction map for clinical kinase inhibitors. *Nature Biotechnology* **2005,** *23* (3), 329-336.
6. Huang, B. X.; Akbar, M.; Kevala, K.; Kim, H. Y., Phosphatidylserine is a critical modulator for Akt activation. *J Cell Biol* **2011,** *192* (6), 979-92.
7. Hiromura, M.; Okada, F.; Obata, T.; Auguin, D.; Shibata, T.; Roumestand, C.; Noguchi, M., Inhibition of Akt kinase activity by a peptide spanning the beta A strand of the proto-oncogene TCL1. *J Biol Chem* **2004,** *279* (51), 53407-53418.

Figures

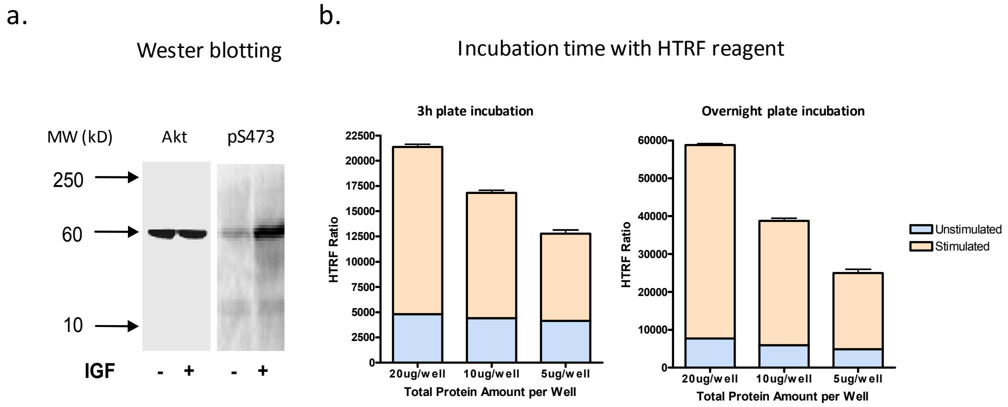

Fig. S1. Akt phosphorylation detected by HTRF assay. The cell lysates were obtained from Neuro 2A cells stimulated with IGF where S473 phosphorylation was increased (a). The HTRF response increased with increasing amounts of cell lysate and incubation time (b). Lysates were incubated with anti-Akt MAb labeled with acceptor fluorophore d2 and anti-pS473-Akt MAb labeled with Europium cryptate. The HTRF ratio represents the ratio of the fluorescence signal at 665nm generated by the acceptor over the fluorescence signal at 620nm produced by the donor, which is indicative of the extent of the S473 phosphorylation. Data represent means ± SEM (n=3).

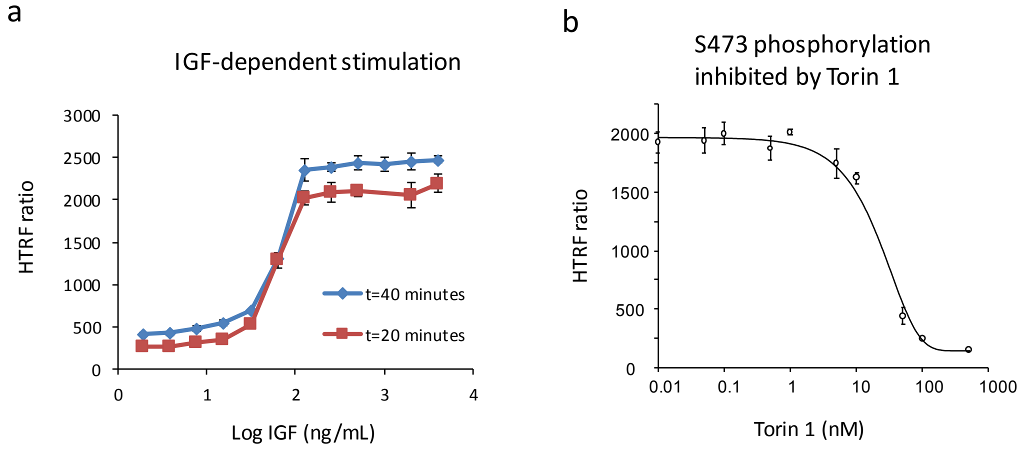

Fig. S2. Development of the HTRF assay in a 384-well format. Neuro 2A cells in 384-well plates (10,000 cells/well) were treated with IGF at different concentrations (2-4000 ng/mL) for 20 or 40 min (a). The HTRF assay was evaluated with Torin 1, an inhibitor known to inhibit mTORC2 activity thus the phosphorylation of Akt S473 (b). The IC50 was determined to be 20 nM. Data represent means ± SEM (n=3).

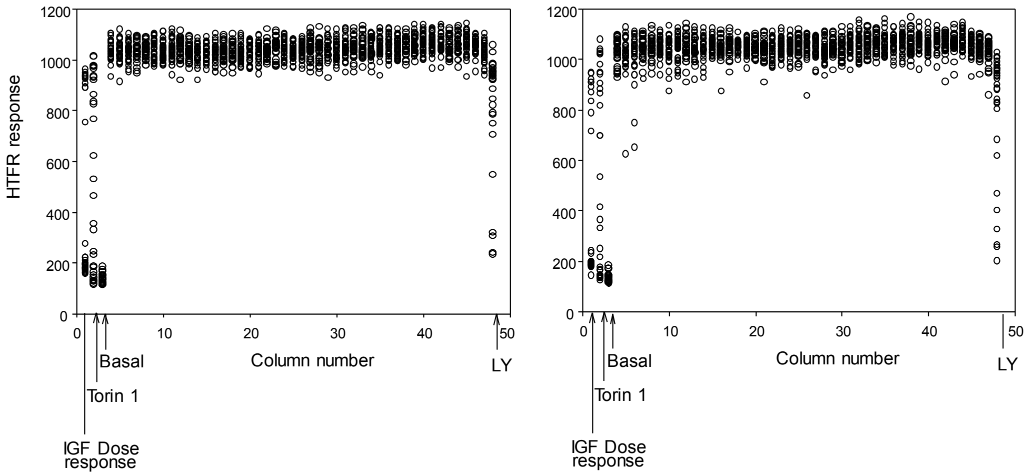

Fig. S3. Evaluation of the HTRF assay by screening the LOPAC library containing 1280 pharmacologically active compounds in a quantitative HTS format. Showing is the readout from two of the 1536-well plates used in the assay. Each compound was diluted at a 1:5 ratio for seven concentrations. The final concentrations of the compounds ranged from 4.90 nM to 76.6 μM. A total of three hits were identified from this screening with a hit rate of 0.24%, demonstrating that this HTRF assay is suitable for HTS. LY, LY294002.

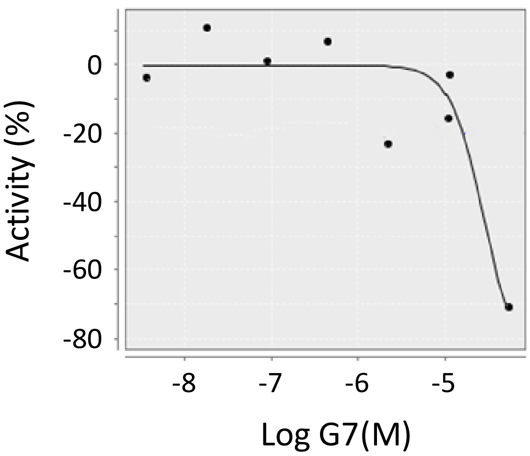

Fig. S4. The HTRF curve of 4-phenylquinolin-2(1H)-one (G7). The IC50 was determined to be ~20 μM.

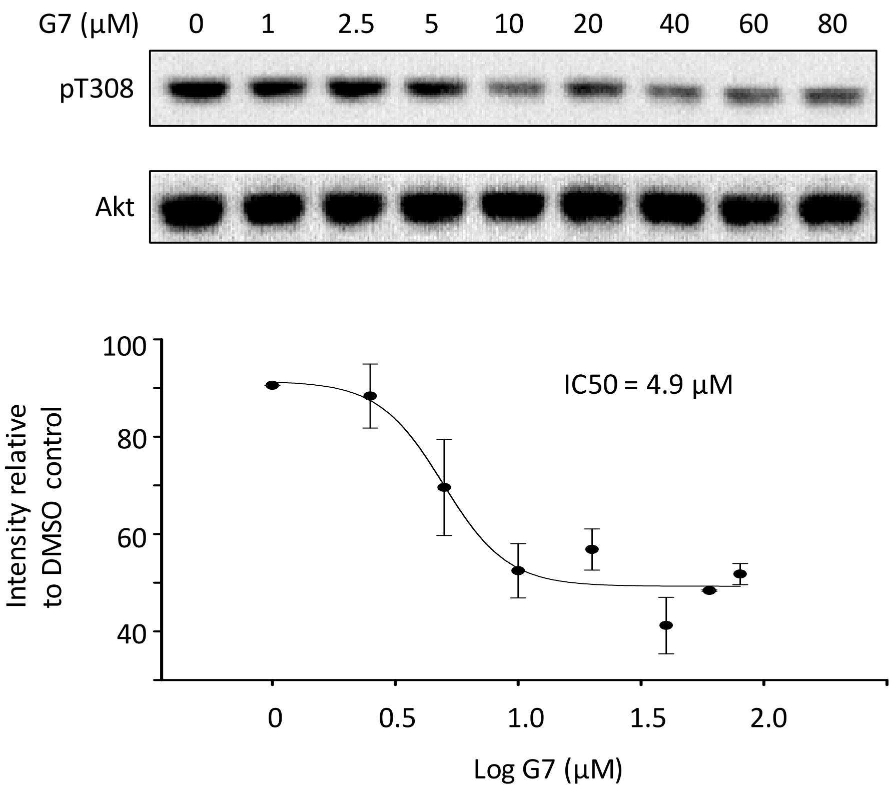

Fig. S5. Evaluation of inhibitor G7 on phosphorylation of T308 by western blot analysis. Neuro 2A cells were incubated with G7 at various concentrations for 30 min followed by IGF stimulation for 30 min. Cells were lysed and subjected to western blot analysis. Data represent means ± SEM of three independent experiments. G7, 4-phenylquinolin-2(1H)-one.

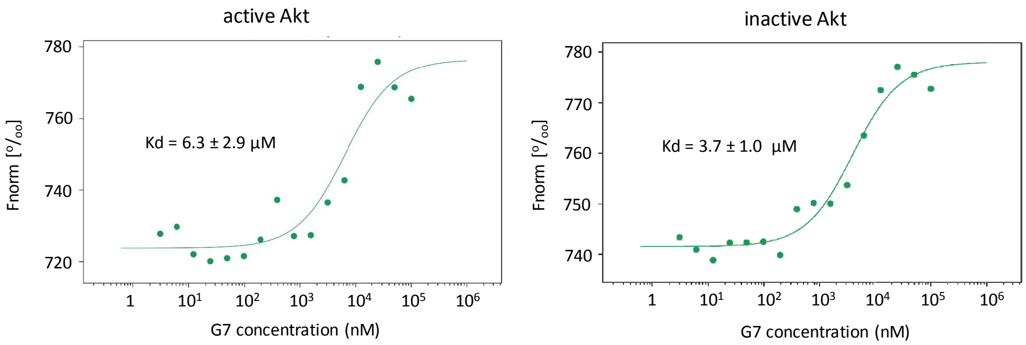

Fig. S6. Binding of G7 to full-length inactive and active Akt assessed by microscale thermophoresis. G7, 4-phenylquinolin-2(1H)-one.

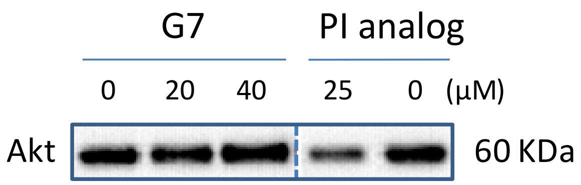

Fig. S7. Effect of G7 on the association of Akt with phosphatidylinositol-3,4,5-trisphosphate (PIP3). Inactive Akt was incubated with inhibitor and PIP3 beads subsequently. Akt bound to PIP3 beads was evaluated by immunoblotting. A PI analog which has been known to interfere with Akt-PIP3 interaction was used as a control. Dotted line, lanes loaded with G7- and the PI analog-treated samples in the same gel were juxtaposed. G7, 4-phenylquinolin-2(1H)-one.

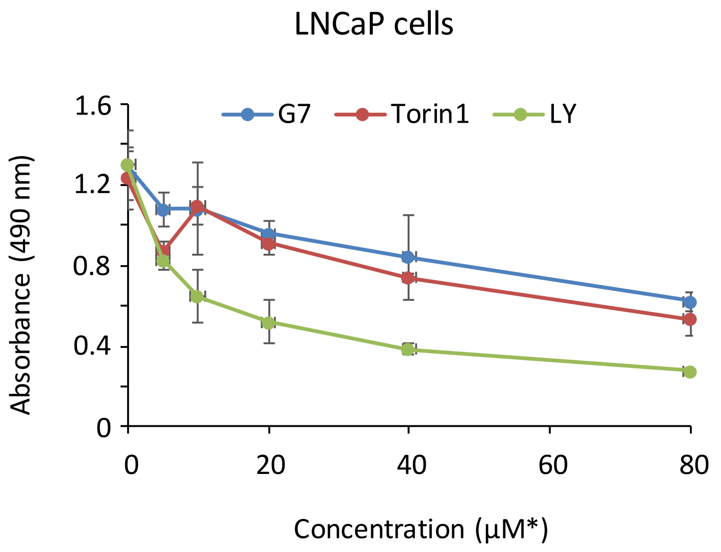

Fig. S8. Effect of G7 on proliferation of LNCaP cells. Cells that were 40% confluent in RPMI-1640 medium containing 10% FBS were treated with inhibitors for 1 day and subjected to MTS assays. *, μM for both G7 and LY and nM for Torin1. Data represent means ± SEM (n=3). G7, 4-phenylquinolin-2(1H)-one, LY, LY294002.

Table S1. Summary of high throughput screening results

Total # compounds screened	373,868
Hits from primary screen	1858
Dose response	106
Elisa assay or wester blotiing verification	75
Selectivity screens against PI3K, PDK1 and SGK1	1

Table S2. KINOMEscan™ selectivity screen

Compound Name	DiscoveRx Gene Symbol	Entrez Gene Symbol	Percent Control	Compound Concentration (nM)
G07	ACVR1	ACVR1	100	50000
G07	ACVR1B	ACVR1B	100	50000
G07	ACVR2A	ACVR2A	100	50000
G07	ACVR2B	ACVR2B	100	50000
G07	ACVRL1	ACVRL1	100	50000
G07	AKT1	AKT1	100	50000
G07	AKT3	AKT3	100	50000
G07	BMPR1A	BMPR1A	100	50000
G07	CAMK4	CAMK4	100	50000
G07	CDC2L1	CDK11B	100	50000
G07	CDK4	CDK4	100	50000
G07	CDKL1	CDKL1	100	50000
G07	CDKL5	CDKL5	100	50000
G07	CHEK2	CHEK2	100	50000
G07	DMPK	DMPK	100	50000
G07	DMPK2	CDC42BPG	100	50000
G07	EGFR(G719S)	EGFR	100	50000
G07	EGFR(T790M)	EGFR	100	50000
G07	EIF2AK1	EIF2AK1	100	50000
G07	EPHA2	EPHA2	100	50000
G07	ERK5	MAPK7	100	50000
G07	GRK3	ADRBK2	100	50000
G07	HCK	HCK	100	50000
G07	IKK-epsilon	IKBKE	100	50000
G07	LIMK1	LIMK1	100	50000
G07	MAP3K4	MAP3K4	100	50000
G07	MAP4K2	MAP4K2	100	50000
G07	MAP4K4	MAP4K4	100	50000
G07	MAP4K5	MAP4K5	100	50000
G07	MAPKAPK2	MAPKAPK2	100	50000
G07	MAST1	MAST1	100	50000
G07	MKK7	MAP2K7	100	50000
G07	MLK2	MAP3K10	100	50000
G07	MRCKA	CDC42BPA	100	50000
G07	MST1R	MST1R	100	50000
G07	MTOR	MTOR	100	50000
G07	MYLK2	MYLK2	100	50000
G07	MYO3B	MYO3B	100	50000
G07	NEK5	NEK5	100	50000

G07	p38-delta	MAPK13	100	50000
G07	p38-gamma	MAPK12	100	50000
G07	PIK3C2B	PIK3C2B	100	50000
G07	PIK3CA(C420R)	PIK3CA	100	50000
G07	PIK3CA(E545A)	PIK3CA	100	50000
G07	PIK3CA(H1047Y)	PIK3CA	100	50000
G07	PIK3CA(M1043I)	PIK3CA	100	50000
G07	PIK3CD	PIK3CD	100	50000
G07	PIP5K2B	PIP4K2B	100	50000
G07	PLK1	PLK1	100	50000
G07	PRKCD	PRKCD	100	50000
G07	PRKCE	PRKCE	100	50000
G07	PRKG2	PRKG2	100	50000
G07	RIPK1	RIPK1	100	50000
G07	RSK2(Kin.Dom.2-C-terminal)	RPS6KA3	100	50000
G07	SgK110	SgK110	100	50000
G07	SRPK3	SRPK3	100	50000
G07	STK33	STK33	100	50000
G07	TAOK3	TAOK3	100	50000
G07	TESK1	TESK1	100	50000
G07	TGFBR1	TGFBR1	100	50000
G07	TGFBR2	TGFBR2	100	50000
G07	TIE2	TEK	100	50000
G07	TNK1	TNK1	100	50000
G07	WEE1	WEE1	100	50000
G07	YANK1	STK32A	100	50000
G07	YANK2	STK32B	100	50000
G07	ZAK	ZAK	100	50000
G07	ZAP70	ZAP70	100	50000
G07	CDK9	CDK9	99	50000
G07	GCN2(Kin.Dom.2,S808G)	EIF2AK4	99	50000
G07	PCTK2	CDK17	99	50000
G07	TNK2	TNK2	99	50000
G07	ERBB2	ERBB2	98	50000
G07	FGFR3(G697C)	FGFR3	98	50000
G07	HIPK4	HIPK4	98	50000
G07	HPK1	MAP4K1	98	50000
G07	PIK3CA(E545K)	PIK3CA	98	50000
G07	PRKCI	PRKCI	98	50000
G07	ROS1	ROS1	98	50000
G07	WNK3	WNK3	98	50000
G07	AMPK-alpha2	PRKAA2	97	50000
G07	DCAMKL2	DCLK2	97	50000
G07	LIMK2	LIMK2	97	50000

G07	MELK	MELK	97	50000
G07	p38-beta	MAPK11	97	50000
G07	PIK3C2G	PIK3C2G	97	50000
G07	WNK1	WNK1	97	50000
G07	ADCK4	ADCK4	96	50000
G07	BRK	PTK6	96	50000
G07	EPHB3	EPHB3	96	50000
G07	FGFR4	FGFR4	96	50000
G07	GRK4	GRK4	96	50000
G07	LKB1	STK11	96	50000
G07	MET(M1250T)	MET	96	50000
G07	PCTK3	CDK18	96	50000
G07	PRKD1	PRKD1	96	50000
G07	S6K1	RPS6KB1	96	50000
G07	ALK	ALK	95	50000
G07	CDK4-cyclinD1	CDK4	95	50000
G07	EPHA5	EPHA5	95	50000
G07	LTK	LTK	95	50000
G07	NEK4	NEK4	95	50000
G07	PIK3CA(I800L)	PIK3CA	95	50000
G07	RAF1	RAF1	95	50000
G07	RIOK1	RIOK1	95	50000
G07	STK36	STK36	95	50000
G07	SYK	SYK	95	50000
G07	TAOK1	TAOK1	95	50000
G07	TBK1	TBK1	95	50000
G07	TNNI3K	TNNI3K	95	50000
G07	TSSK3	TSSK3	95	50000
G07	ABL1(F317L)-nonphosphorylated	ABL1	94	50000
G07	ABL2	ABL2	94	50000
G07	ASK1	MAP3K5	94	50000
G07	LZK	MAP3K13	94	50000
G07	PFTK1	CDK14	94	50000
G07	PIK3CG	PIK3CG	94	50000
G07	AKT2	AKT2	93	50000
G07	EPHA4	EPHA4	93	50000
G07	EPHA8	EPHA8	93	50000
G07	EPHB2	EPHB2	93	50000
G07	ERK2	MAPK1	93	50000
G07	FAK	PTK2	93	50000
G07	MEK4	MAP2K4	93	50000
G07	NIK	MAP3K14	93	50000
G07	PYK2	PTK2B	93	50000
G07	RPS6KA5(Kin.Dom.2-C-terminal)	RPS6KA5	93	50000

G07	TAOK2	TAOK2	93	50000
G07	TSSK1B	TSSK1B	93	50000
G07	TXK	TXK	93	50000
G07	TYRO3	TYRO3	93	50000
G07	YANK3	STK32C	93	50000
G07	YES	YES1	93	50000
G07	ADCK3	CABC1	92	50000
G07	CAMK2B	CAMK2B	92	50000
G07	CLK3	CLK3	92	50000
G07	EPHB4	EPHB4	92	50000
G07	ERK3	MAPK6	92	50000
G07	FES	FES	92	50000
G07	IKK-alpha	CHUK	92	50000
G07	NEK6	NEK6	92	50000
G07	RPS6KA4(Kin.Dom.2-C-terminal)	RPS6KA4	92	50000
G07	AURKB	AURKB	91	50000
G07	CDK11	CDK19	91	50000
G07	DCAMKL3	DCLK3	91	50000
G07	EPHB1	EPHB1	91	50000
G07	GRK2	ADRBK1	91	50000
G07	PCTK1	CDK16	91	50000
G07	PHKG2	PHKG2	91	50000
G07	AMPK-alpha1	PRKAA1	90	50000
G07	CAMKK2	CAMKK2	90	50000
G07	EGFR(L858R,T790M)	EGFR	90	50000
G07	MST1	STK4	90	50000
G07	MYLK	MYLK	90	50000
G07	NEK11	NEK11	90	50000
G07	NIM1	MGC42105	90	50000
G07	STK35	STK35	90	50000
G07	CASK	CASK	89	50000
G07	CDKL3	CDKL3	89	50000
G07	CSNK1G1	CSNK1G1	89	50000
G07	EGFR	EGFR	89	50000
G07	EGFR(L747-E749del, A750P)	EGFR	89	50000
G07	EPHA1	EPHA1	89	50000
G07	EPHA7	EPHA7	89	50000
G07	ERK1	MAPK3	89	50000
G07	FRK	FRK	89	50000
G07	FYN	FYN	89	50000
G07	MAP4K3	MAP4K3	89	50000
G07	PIK3CA(H1047L)	PIK3CA	89	50000
G07	PKN1	PKN1	89	50000
G07	PKN2	PKN2	89	50000

G07	PRKD3	PRKD3	89	50000
G07	RPS6KA5(Kin.Dom.1-N-terminal)	RPS6KA5	89	50000
G07	SLK	SLK	89	50000
G07	TLK2	TLK2	89	50000
G07	CDC2L2	CDC2L2	88	50000
G07	CDK2	CDK2	88	50000
G07	CDK3	CDK3	88	50000
G07	EPHA6	EPHA6	88	50000
G07	ERBB3	ERBB3	88	50000
G07	FGR	FGR	88	50000
G07	LYN	LYN	88	50000
G07	MARK4	MARK4	88	50000
G07	MLK3	MAP3K11	88	50000
G07	NDR2	STK38L	88	50000
G07	NEK1	NEK1	88	50000
G07	SRMS	SRMS	88	50000
G07	WNK4	WNK4	88	50000
G07	CAMK1D	CAMK1D	87	50000
G07	CAMK2A	CAMK2A	87	50000
G07	CDC2L5	CDK13	87	50000
G07	DCAMKL1	DCLK1	87	50000
G07	DDR2	DDR2	87	50000
G07	LATS1	LATS1	87	50000
G07	MARK3	MARK3	87	50000
G07	OSR1	OXSR1	87	50000
G07	PAK6	PAK6	87	50000
G07	PKAC-alpha	PRKACA	87	50000
G07	ABL1(T315I)-nonphosphorylated	ABL1	86	50000
G07	BMX	BMX	86	50000
G07	DRAK2	STK17B	86	50000
G07	ERBB4	ERBB4	86	50000
G07	ERK4	MAPK4	86	50000
G07	JNK3	MAPK10	86	50000
G07	PKAC-beta	PRKACB	86	50000
G07	SBK1	SBK1	86	50000
G07	ULK3	ULK3	86	50000
G07	ALK(L1196M)	ALK	85	50000
G07	BUB1	BUB1	85	50000
G07	CDK8	CDK8	85	50000
G07	CHEK1	CHEK1	85	50000
G07	EGFR(S752-I759del)	EGFR	85	50000
G07	MYO3A	MYO3A	85	50000
G07	PFPK5(P.falciparum)	MAL13P1.279	85	50000
G07	QSK	KIAA0999	85	50000

G07	RET(V804M)	RET	85	50000
G07	STK39	STK39	85	50000
G07	BRAF	BRAF	84	50000
G07	CAMK1	CAMK1	84	50000
G07	CDK7	CDK7	84	50000
G07	DDR1	DDR1	84	50000
G07	EGFR(L747-S752del, P753S)	EGFR	84	50000
G07	FER	FER	84	50000
G07	KIT(D816H)	KIT	84	50000
G07	MRCKB	CDC42BPB	84	50000
G07	PAK4	PAK4	84	50000
G07	SNRK	SNRK	84	50000
G07	SRPK2	SRPK2	84	50000
G07	TIE1	TIE1	84	50000
G07	WEE2	WEE2	84	50000
G07	WNK2	WNK2	84	50000
G07	BMPR1B	BMPR1B	83	50000
G07	DAPK2	DAPK2	83	50000
G07	EGFR(L858R)	EGFR	83	50000
G07	FGFR3	FGFR3	83	50000
G07	IKK-beta	IKBKB	83	50000
G07	NDR1	STK38	83	50000
G07	p38-alpha	MAPK14	83	50000
G07	PFCDPK1(P.falciparum)	CDPK1	83	50000
G07	PIK3CA(Q546K)	PIK3CA	83	50000
G07	PIP5K2C	PIP4K2C	83	50000
G07	PLK3	PLK3	83	50000
G07	RET(V804L)	RET	83	50000
G07	RIOK2	RIOK2	83	50000
G07	SGK3	SGK3	83	50000
G07	BMPR2	BMPR2	82	50000
G07	BRSK2	BRSK2	82	50000
G07	JAK1(JH1domain-catalytic)	JAK1	82	50000
G07	LOK	STK10	82	50000
G07	MLK1	MAP3K9	82	50000
G07	NLK	NLK	82	50000
G07	PAK1	PAK1	82	50000
G07	PIK3CA	PIK3CA	82	50000
G07	PIK3CA(E542K)	PIK3CA	82	50000
G07	PRKD2	PRKD2	82	50000
G07	RIPK2	RIPK2	82	50000
G07	VRK2	VRK2	82	50000
G07	ABL1-nonphosphorylated	ABL1	81	50000
G07	CAMK2G	CAMK2G	81	50000

G07	CSNK1A1L	CSNK1A1L	81	50000
G07	FLT1	FLT1	81	50000
G07	MAPKAPK5	MAPKAPK5	81	50000
G07	MEK3	MAP2K3	81	50000
G07	PAK3	PAK3	81	50000
G07	PDPK1	PDPK1	81	50000
G07	PFTAIRE2	CDK15	81	50000
G07	PRKCQ	PRKCQ	81	50000
G07	ROCK2	ROCK2	81	50000
G07	ALK(C1156Y)	ALK	80	50000
G07	CAMKK1	CAMKK1	80	50000
G07	CLK4	CLK4	80	50000
G07	CSNK1A1	CSNK1A1	80	50000
G07	FGFR2	FGFR2	80	50000
G07	LCK	LCK	80	50000
G07	NEK9	NEK9	80	50000
G07	PLK4	PLK4	80	50000
G07	RIPK4	RIPK4	80	50000
G07	BLK	BLK	79	50000
G07	CAMK2D	CAMK2D	79	50000
G07	EGFR(G719C)	EGFR	79	50000
G07	PHKG1	PHKG1	79	50000
G07	PRP4	PRPF4B	79	50000
G07	ASK2	MAP3K6	78	50000
G07	CSNK2A1	CSNK2A1	78	50000
G07	DLK	MAP3K12	78	50000
G07	KIT(A829P)	KIT	78	50000
G07	MLCK	MYLK3	78	50000
G07	NEK7	NEK7	78	50000
G07	PLK2	PLK2	78	50000
G07	SIK2	SIK2	78	50000
G07	TRPM6	TRPM6	78	50000
G07	CAMK1G	CAMK1G	77	50000
G07	EGFR(L747-T751del,Sins)	EGFR	77	50000
G07	KIT-autoinhibited	KIT	77	50000
G07	MAK	MAK	77	50000
G07	MINK	MINK1	77	50000
G07	NEK2	NEK2	77	50000
G07	CSK	CSK	76	50000
G07	DYRK1B	DYRK1B	76	50000
G07	FLT3(R834Q)	FLT3	76	50000
G07	IGF1R	IGF1R	76	50000
G07	MARK1	MARK1	76	50000
G07	MST2	STK3	76	50000

G07	PRKX	PRKX	76	50000
G07	ULK2	ULK2	76	50000
G07	CDK5	CDK5	75	50000
G07	GRK1	GRK1	75	50000
G07	JNK1	MAPK8	75	50000
G07	MUSK	MUSK	75	50000
G07	PRKCH	PRKCH	75	50000
G07	STK16	STK16	75	50000
G07	CIT	CIT	74	50000
G07	CSNK1G2	CSNK1G2	74	50000
G07	CTK	MATK	74	50000
G07	DAPK3	DAPK3	74	50000
G07	HIPK1	HIPK1	74	50000
G07	IRAK3	IRAK3	74	50000
G07	PIKFYVE	PIKFYVE	74	50000
G07	PRKR	EIF2AK2	74	50000
G07	ULK1	ULK1	74	50000
G07	ABL1-phosphorylated	ABL1	73	50000
G07	ANKK1	ANKK1	73	50000
G07	MET	MET	73	50000
G07	RIPK5	DSTYK	73	50000
G07	ABL1(F317I)-phosphorylated	ABL1	72	50000
G07	BRSK1	BRSK1	72	50000
G07	DYRK2	DYRK2	72	50000
G07	FLT4	FLT4	72	50000
G07	MEK2	MAP2K2	72	50000
G07	MEK6	MAP2K6	72	50000
G07	TYK2(JH1domain-catalytic)	TYK2	72	50000
G07	ABL1(F317I)-nonphosphorylated	ABL1	71	50000
G07	BRAF(V600E)	BRAF	71	50000
G07	CDK4-cyclinD3	CDK4	71	50000
G07	CSNK1G3	CSNK1G3	71	50000
G07	EGFR(L861Q)	EGFR	71	50000
G07	ICK	ICK	71	50000
G07	MAP3K1	MAP3K1	71	50000
G07	MAP3K15	MAP3K15	71	50000
G07	TEC	TEC	71	50000
G07	CSNK2A2	CSNK2A2	70	50000
G07	EGFR(E746-A750del)	EGFR	70	50000
G07	HUNK	HUNK	70	50000
G07	MEK5	MAP2K5	70	50000
G07	PIP5K1A	PIP5K1A	70	50000
G07	PRKG1	PRKG1	70	50000
G07	RET(M918T)	RET	70	50000

G07	YSK1	STK25	70	50000
G07	CDKL2	CDKL2	69	50000
G07	CLK1	CLK1	69	50000
G07	ERN1	ERN1	69	50000
G07	MEK1	MAP2K1	69	50000
G07	MKNK1	MKNK1	69	50000
G07	PIK3CB	PIK3CB	69	50000
G07	ROCK1	ROCK1	69	50000
G07	RSK3(Kin.Dom.1-N-terminal)	RPS6KA2	69	50000
G07	GSK3B	GSK3B	68	50000
G07	IRAK4	IRAK4	68	50000
G07	JNK2	MAPK9	68	50000
G07	NEK3	NEK3	68	50000
G07	PAK2	PAK2	68	50000
G07	PIM2	PIM2	68	50000
G07	VEGFR2	KDR	67	50000
G07	ABL1(H396P)-phosphorylated	ABL1	66	50000
G07	AURKC	AURKC	66	50000
G07	EPHA3	EPHA3	66	50000
G07	NEK10	NEK10	66	50000
G07	SGK	SGK1	66	50000
G07	DRAK1	STK17A	65	50000
G07	HIPK3	HIPK3	65	50000
G07	MARK2	MARK2	65	50000
G07	MKNK2	MKNK2	65	50000
G07	PKMYT1	PKMYT1	65	50000
G07	SIK	SIK1	65	50000
G07	SRC	SRC	65	50000
G07	TRKA	NTRK1	65	50000
G07	ABL1(F317L)-phosphorylated	ABL1	64	50000
G07	ABL1(T315I)-phosphorylated	ABL1	64	50000
G07	CLK2	CLK2	64	50000
G07	INSRR	INSRR	64	50000
G07	PIK4CB	PI4KB	64	50000
G07	PIM3	PIM3	64	50000
G07	DAPK1	DAPK1	63	50000
G07	HIPK2	HIPK2	63	50000
G07	LRRK2(G2019S)	LRRK2	63	50000
G07	MAP3K2	MAP3K2	63	50000
G07	AAK1	AAK1	62	50000
G07	ABL1(H396P)-nonphosphorylated	ABL1	62	50000
G07	MAP3K3	MAP3K3	62	50000
G07	SGK2	SGK2	62	50000
G07	JAK2(JH1domain-catalytic)	JAK2	61	50000

G07	RPS6KA4(Kin.Dom.1-N-terminal)	RPS6KA4	61	50000
G07	TAK1	MAP3K7	61	50000
G07	TRKC	NTRK3	61	50000
G07	ITK	ITK	60	50000
G07	TNIK	TNIK	60	50000
G07	ARK5	NUAK1	59	50000
G07	MET(Y1235D)	MET	59	50000
G07	MST3	STK24	59	50000
G07	RSK3(Kin.Dom.2-C-terminal)	RPS6KA2	59	50000
G07	TLK1	TLK1	59	50000
G07	KIT(D816V)	KIT	58	50000
G07	RIOK3	RIOK3	58	50000
G07	HASPIN	GSG2	57	50000
G07	PAK7	PAK7	57	50000
G07	ABL1(E255K)-phosphorylated	ABL1	56	50000
G07	ABL1(Y253F)-phosphorylated	ABL1	56	50000
G07	FGFR1	FGFR1	56	50000
G07	GAK	GAK	56	50000
G07	INSR	INSR	56	50000
G07	JAK3(JH1domain-catalytic)	JAK3	56	50000
G07	TRKB	NTRK2	56	50000
G07	ABL1(M351T)-phosphorylated	ABL1	55	50000
G07	LRRK2	LRRK2	55	50000
G07	YSK4	MAP3K19	55	50000
G07	DYRK1A	DYRK1A	54	50000
G07	FLT3-autoinhibited	FLT3	54	50000
G07	IRAK1	IRAK1	53	50000
G07	MST4	MST4	53	50000
G07	BIKE	BMP2K	52	50000
G07	CAMK1B	PNCK	52	50000
G07	FLT3(ITD)	FLT3	52	50000
G07	GRK7	GRK7	52	50000
G07	PDGFRA	PDGFRA	52	50000
G07	RET	RET	51	50000
G07	AURKA	AURKA	50	50000
G07	BTK	BTK	50	50000
G07	PIM1	PIM1	49	50000
G07	KIT(V559D,V654A)	KIT	48	50000
G07	SRPK1	SRPK1	47	50000
G07	ABL1(Q252H)-nonphosphorylated	ABL1	46	50000
G07	ERK8	MAPK15	46	50000
G07	CSNK1D	CSNK1D	45	50000
G07	SNARK	NUAK2	45	50000
G07	KIT(V559D,T670I)	KIT	44	50000

G07	PIP5K1C	PIP5K1C	44	50000
G07	MYLK4	MYLK4	43	50000
G07	PKNB(M.tuberculosis)	pknB	43	50000
G07	AXL	AXL	42	50000
G07	MERTK	MERTK	42	50000
G07	FLT3(ITD,F691L)	FLT3	40	50000
G07	RSK1(Kin.Dom.2-C-terminal)	RPS6KA1	39	50000
G07	RSK4(Kin.Dom.2-C-terminal)	RPS6KA6	38	50000
G07	VPS34	PIK3C3	35	50000
G07	FLT3(D835H)	FLT3	34	50000
G07	FLT3(ITD,D835V)	FLT3	34	50000
G07	RSK4(Kin.Dom.1-N-terminal)	RPS6KA6	34	50000
G07	ABL1(Q252H)-phosphorylated	ABL1	33	50000
G07	FLT3(D835Y)	FLT3	33	50000
G07	FLT3(N841I)	FLT3	30	50000
G07	FLT3(K663Q)	FLT3	29	50000
G07	KIT	KIT	26	50000
G07	TTK	TTK	24	50000
G07	LATS2	LATS2	23	50000
G07	PDGFRB	PDGFRB	23	50000
G07	RSK1(Kin.Dom.1-N-terminal)	RPS6KA1	23	50000
G07	GSK3A	GSK3A	22	50000
G07	KIT(L576P)	KIT	22	50000
G07	KIT(V559D)	KIT	20	50000
G07	RSK2(Kin.Dom.1-N-terminal)	RPS6KA3	17	50000
G07	CSF1R-autoinhibited	CSF1R	16	50000
G07	CSNK1E	CSNK1E	15	50000
G07	TYK2(JH2domain-pseudokinase)	TYK2	15	50000
G07	EPHB6	EPHB6	14	50000
G07	FLT3	FLT3	13	50000
G07	FLT3(D835V)	FLT3	7.6	50000
G07	CSF1R	CSF1R	7.4	50000
G07	JAK1(JH2domain-pseudokinase)	JAK1	5.5	50000

Note: A percent control of less than 35% is deemed a significant hit.

Compound Name	Selectivity Score Type	Number of Hits	Number of Non-Mutant Kinases	Screening Concentration (nM)	Selectivity Score
G07	S(35)	14	403	50000	0.035
G07	S(10)	2	403	50000	0.005
G07	S(1)	0	403	50000	0

Fig. S9. Full-length gels and blots

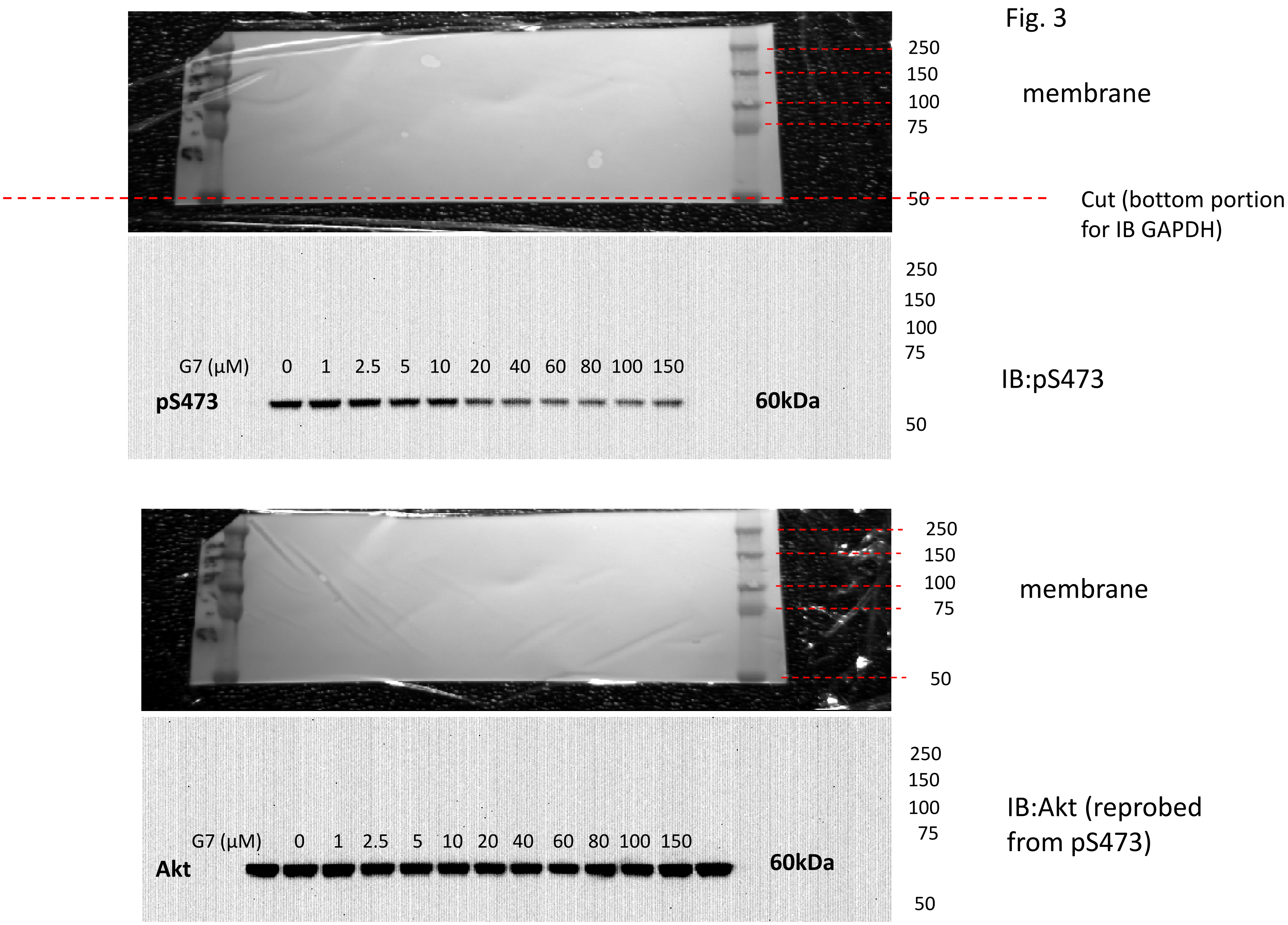

Fig. 4b

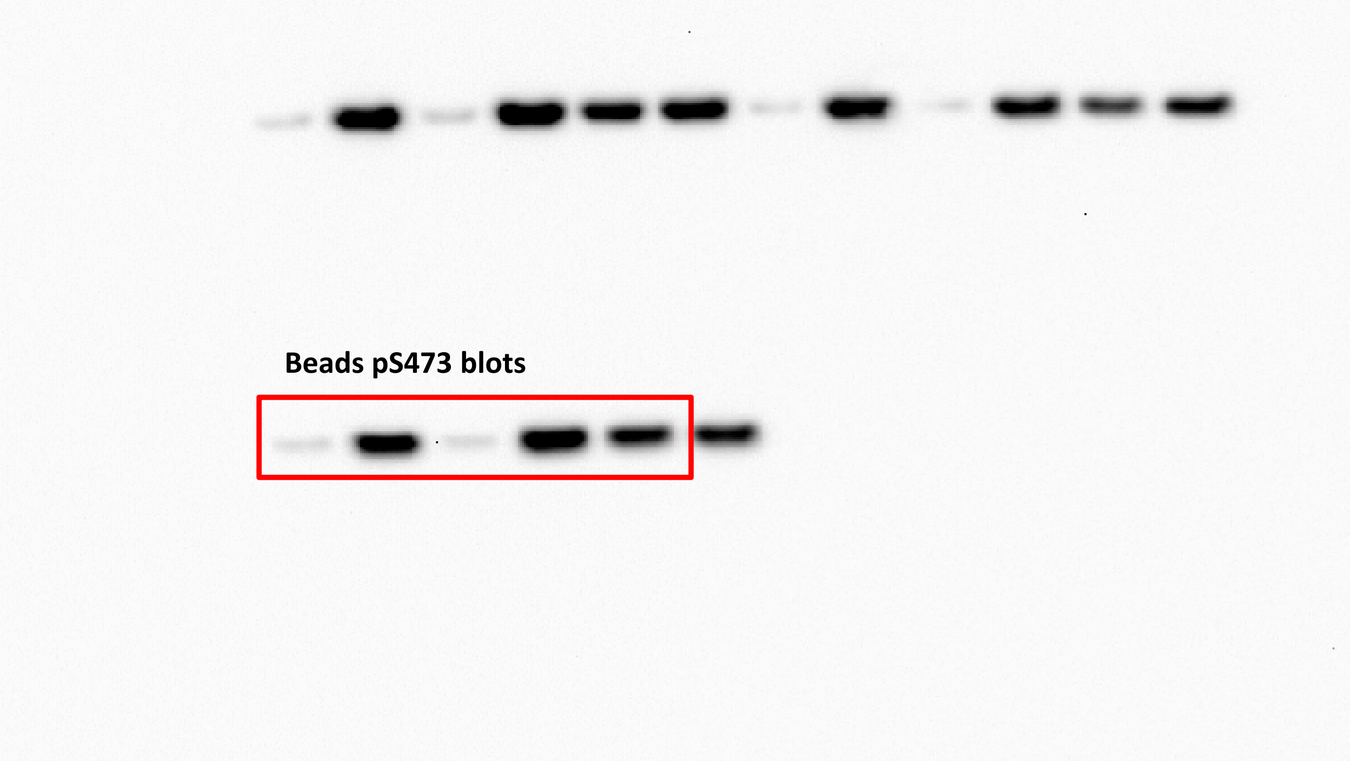

Fig. 4b

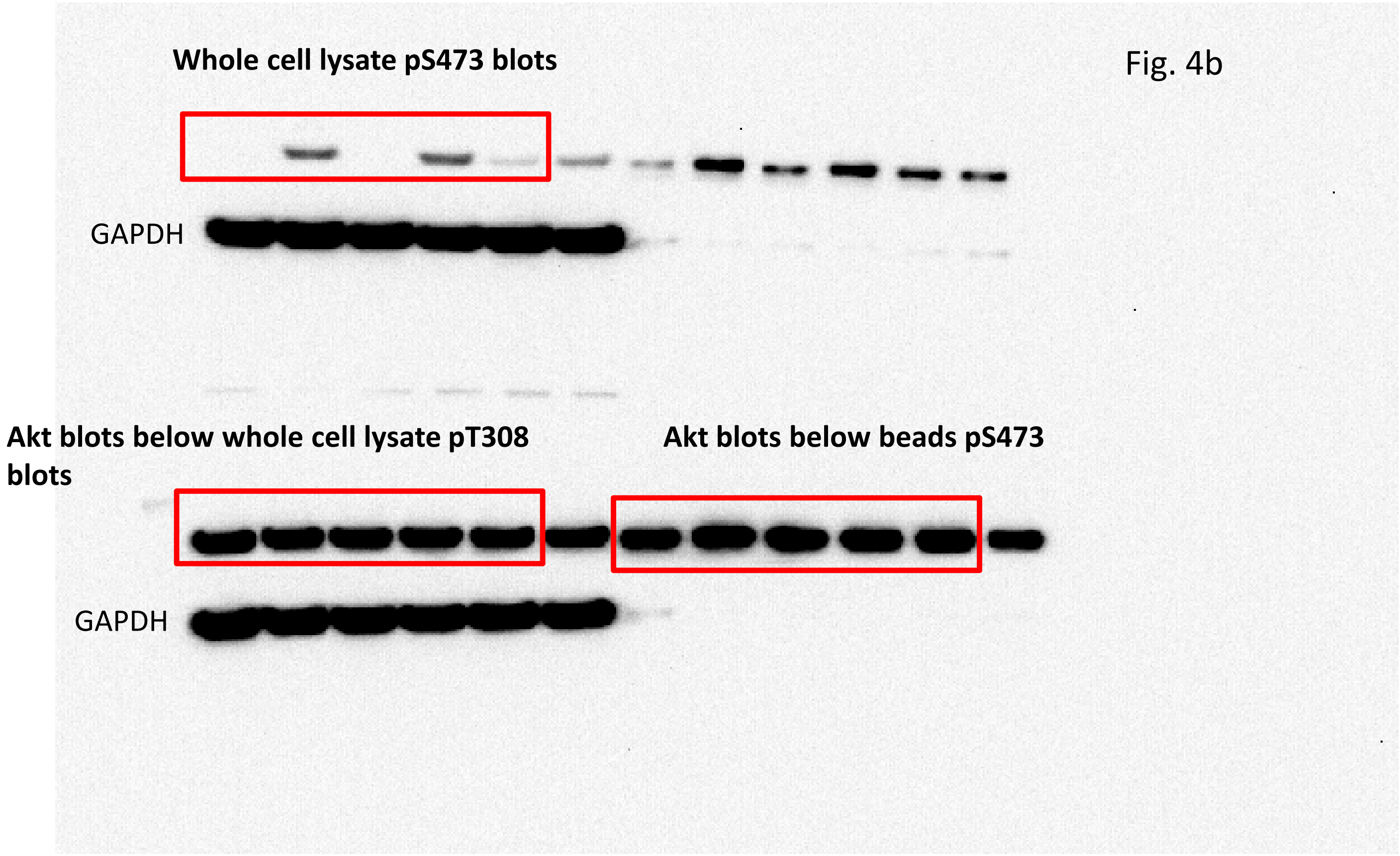
Whole cell lysate pS473 blots
GAPDH
Akt blots below whole cell lysate pT308 blots
Akt blots below beads pS473
GAPDH

Fig. 4b

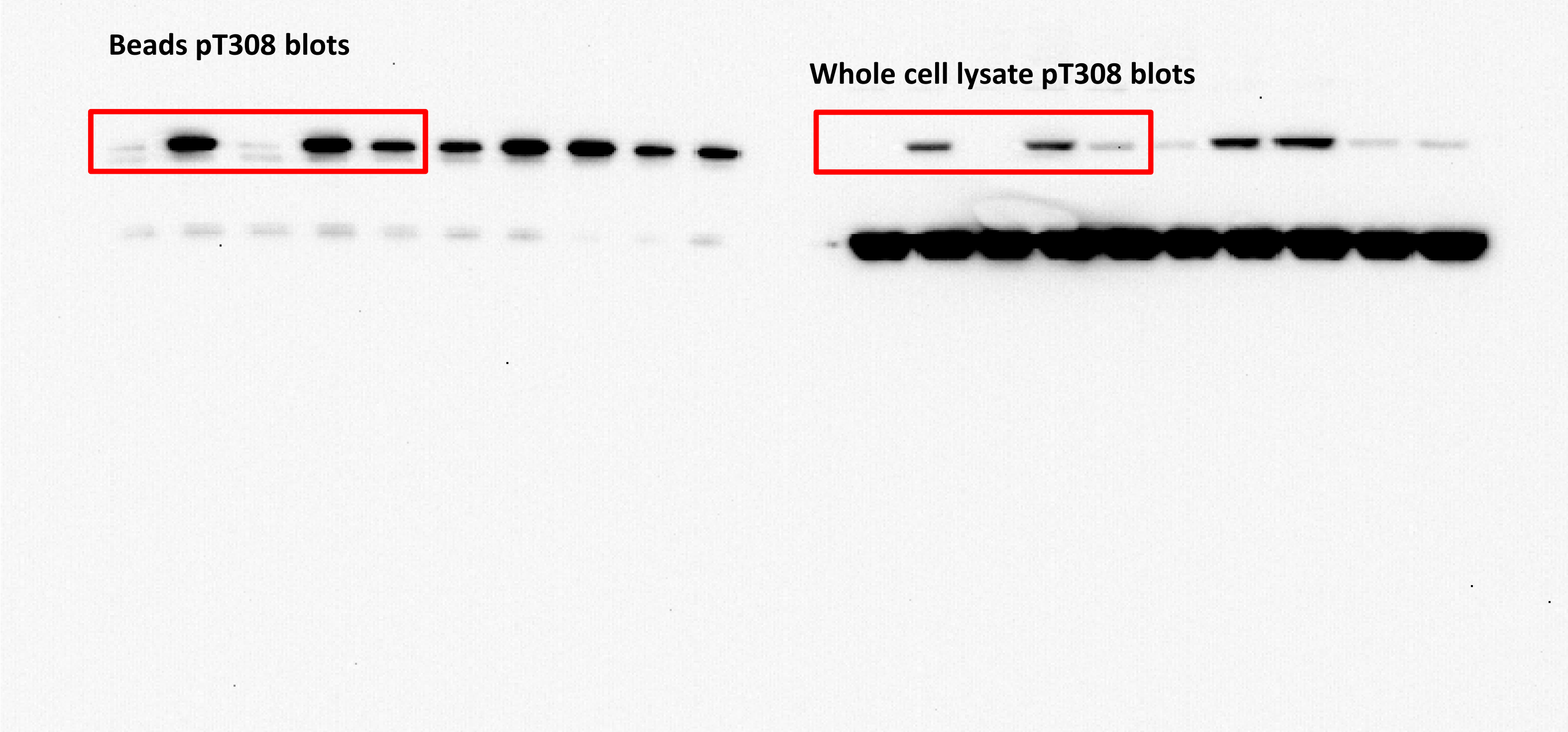

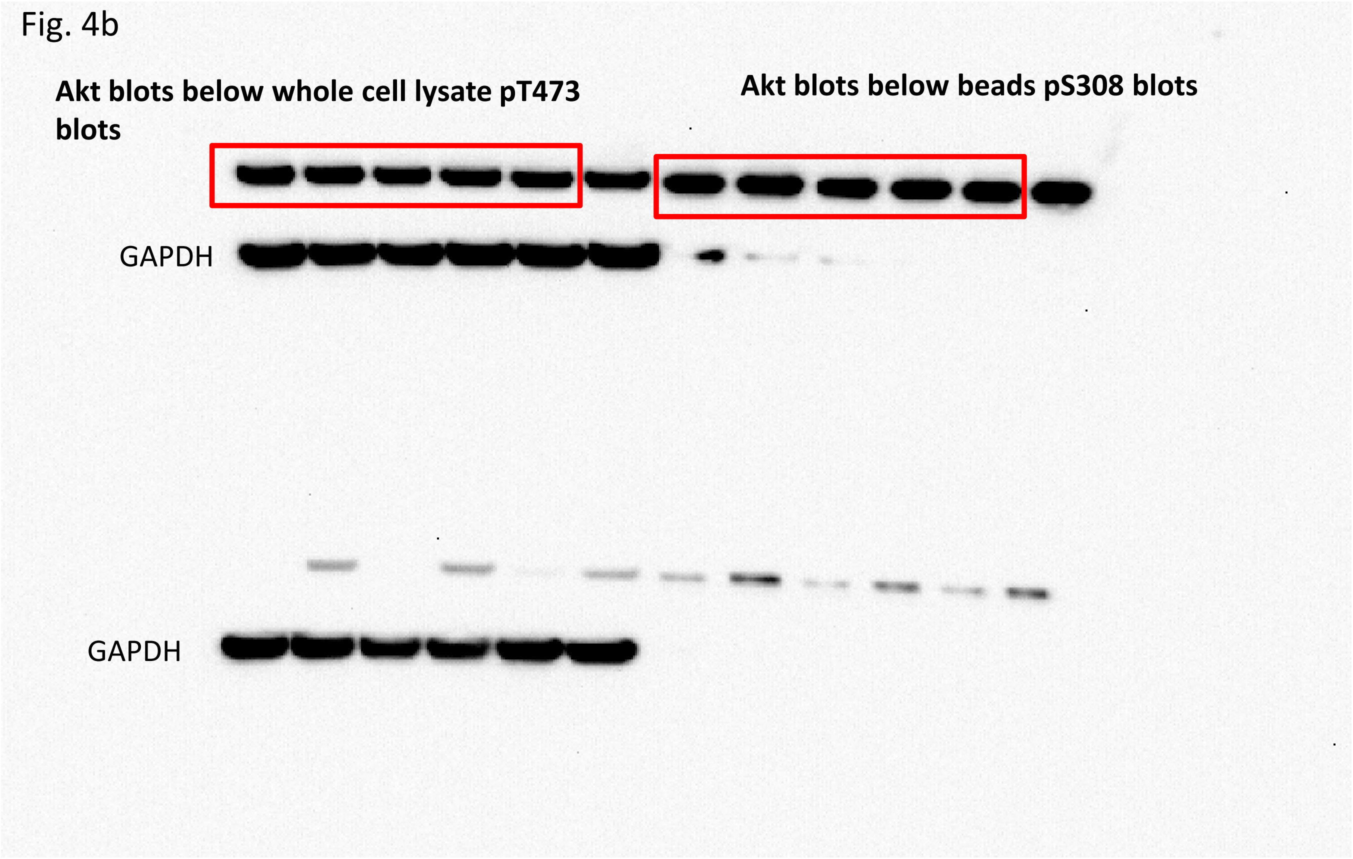
Fig. 4b
Akt blots below whole cell lysate pT473 blots
Akt blots below beads pS308 blots
GAPDH
GAPDH

Example 1 of cutting the membrane for blotting with different antibody

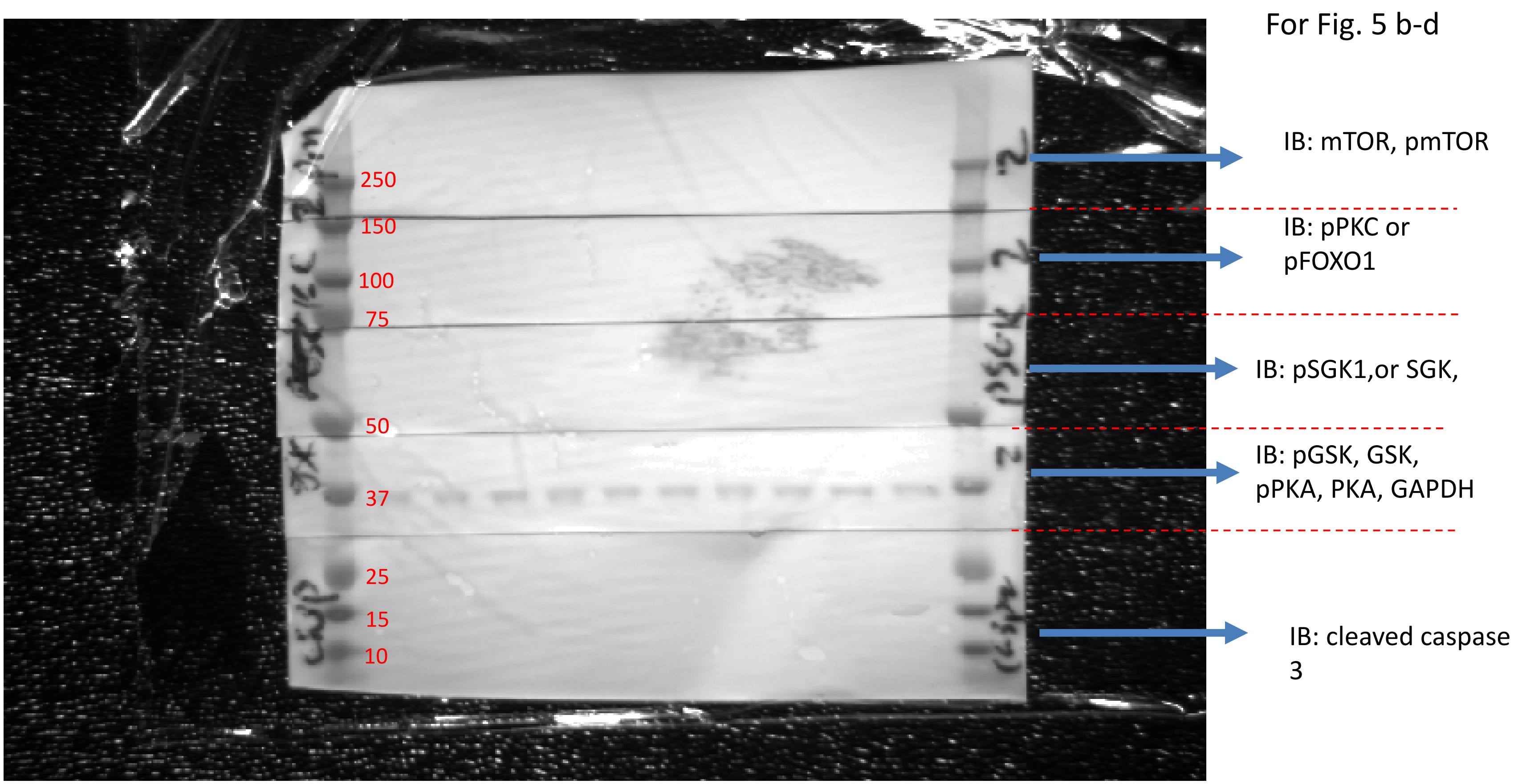

Example 2 of cutting the membrane for blotting with different antibody

For Fig. 5 b-d

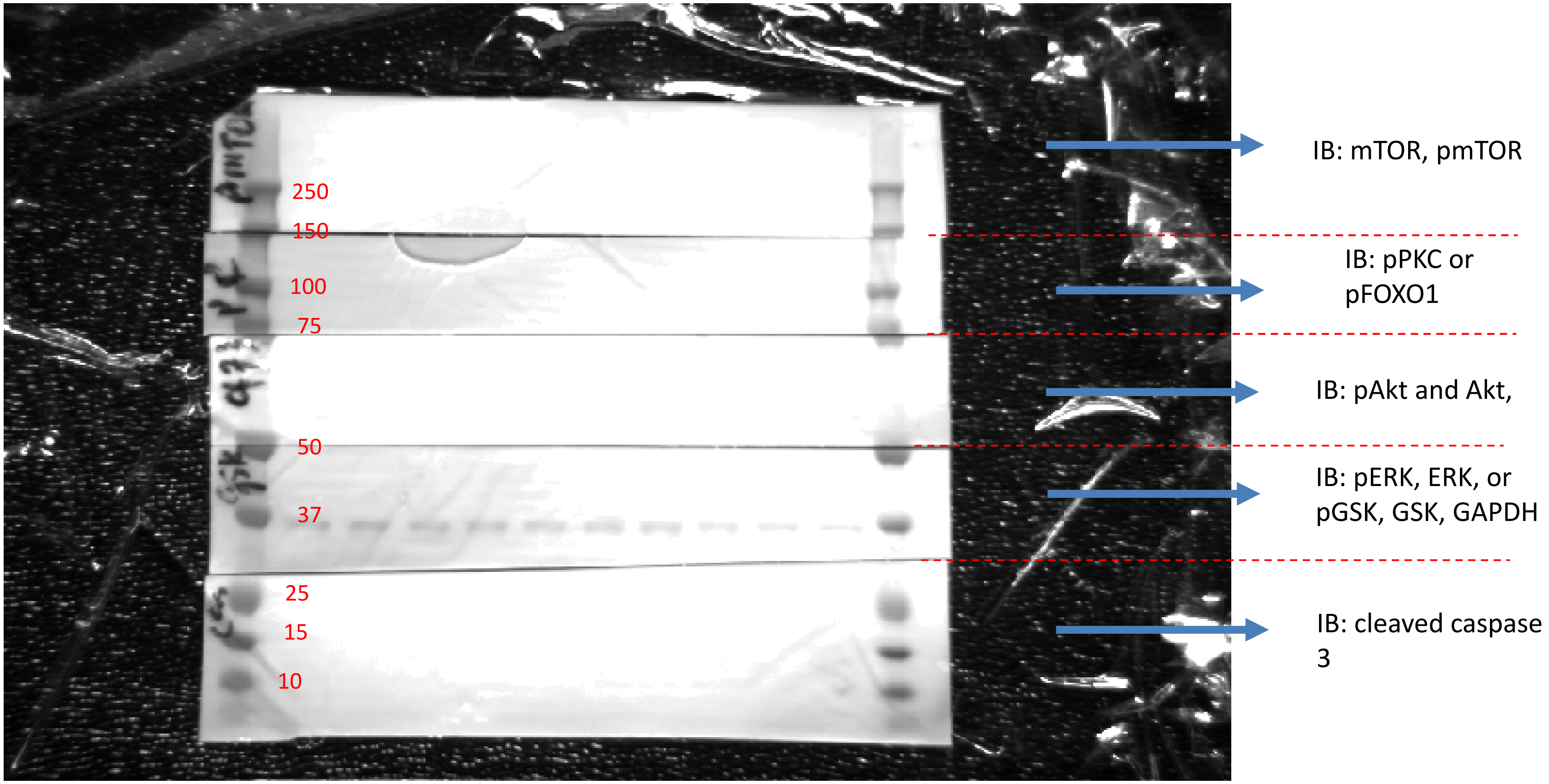

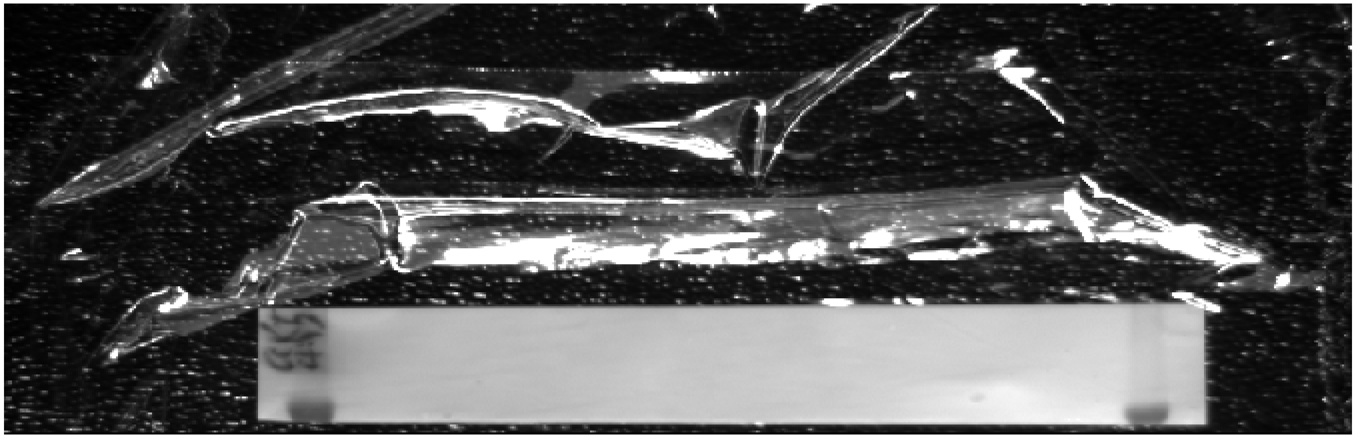

Fig. 5b

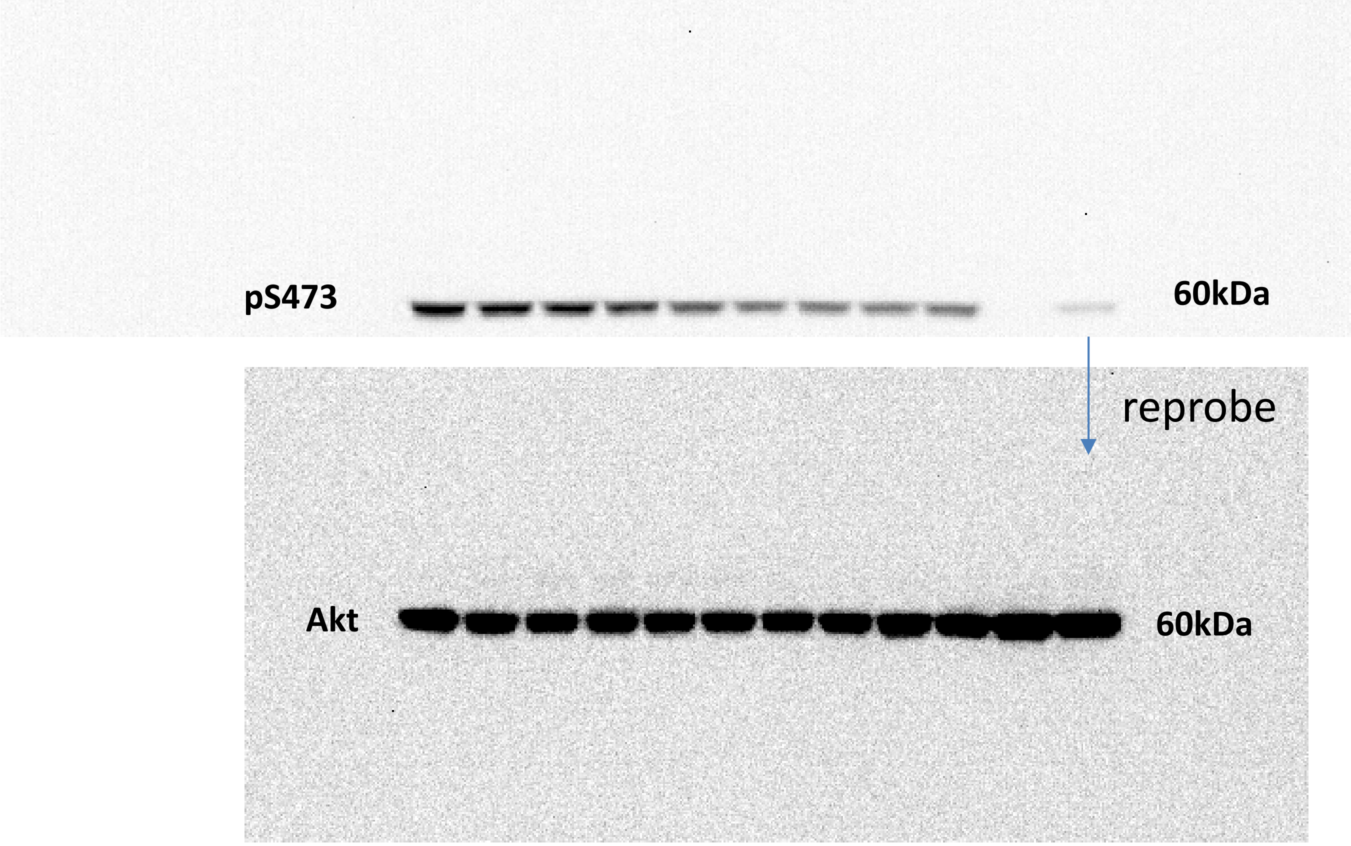

Fig. 5b

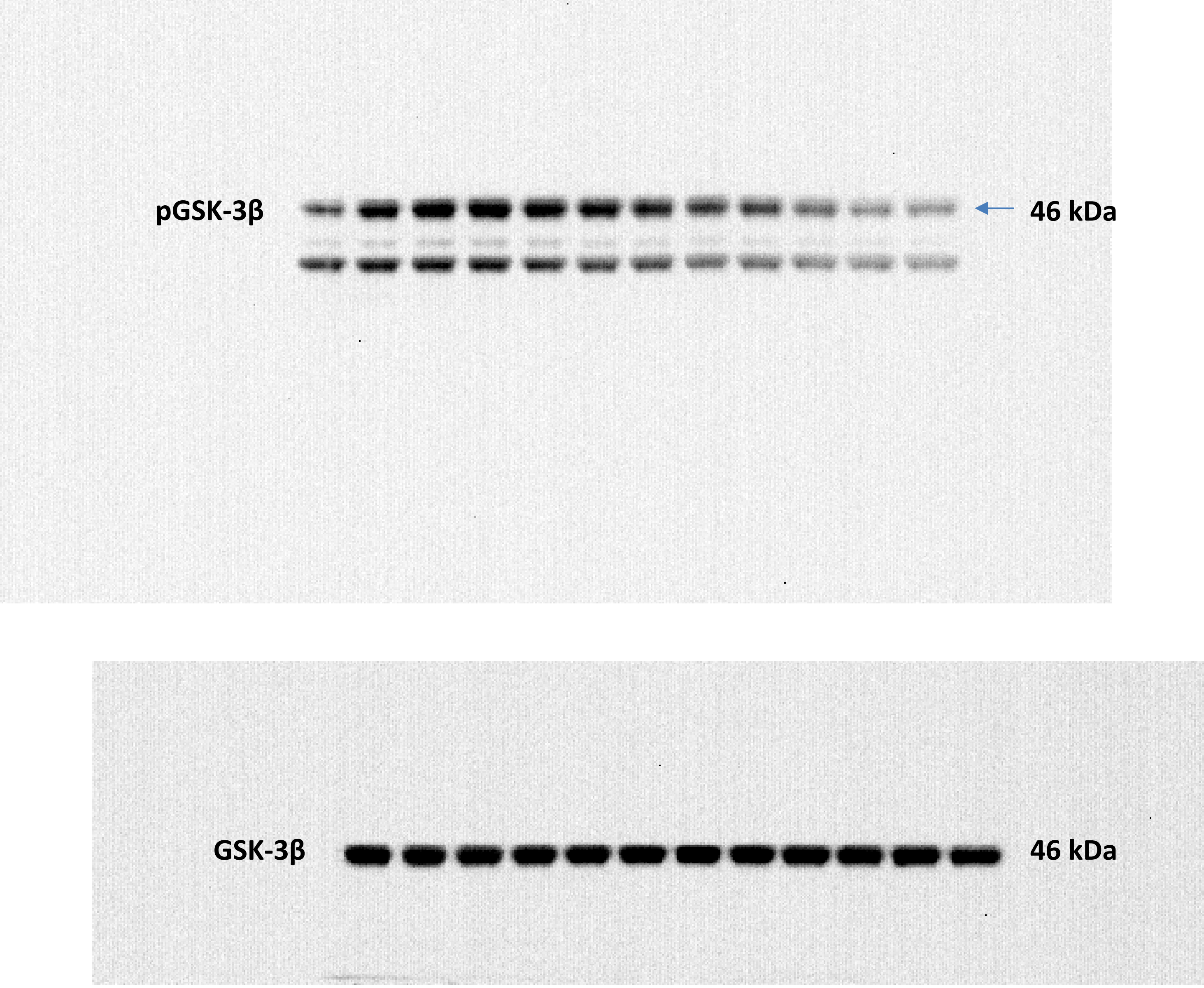

Fig. 5b

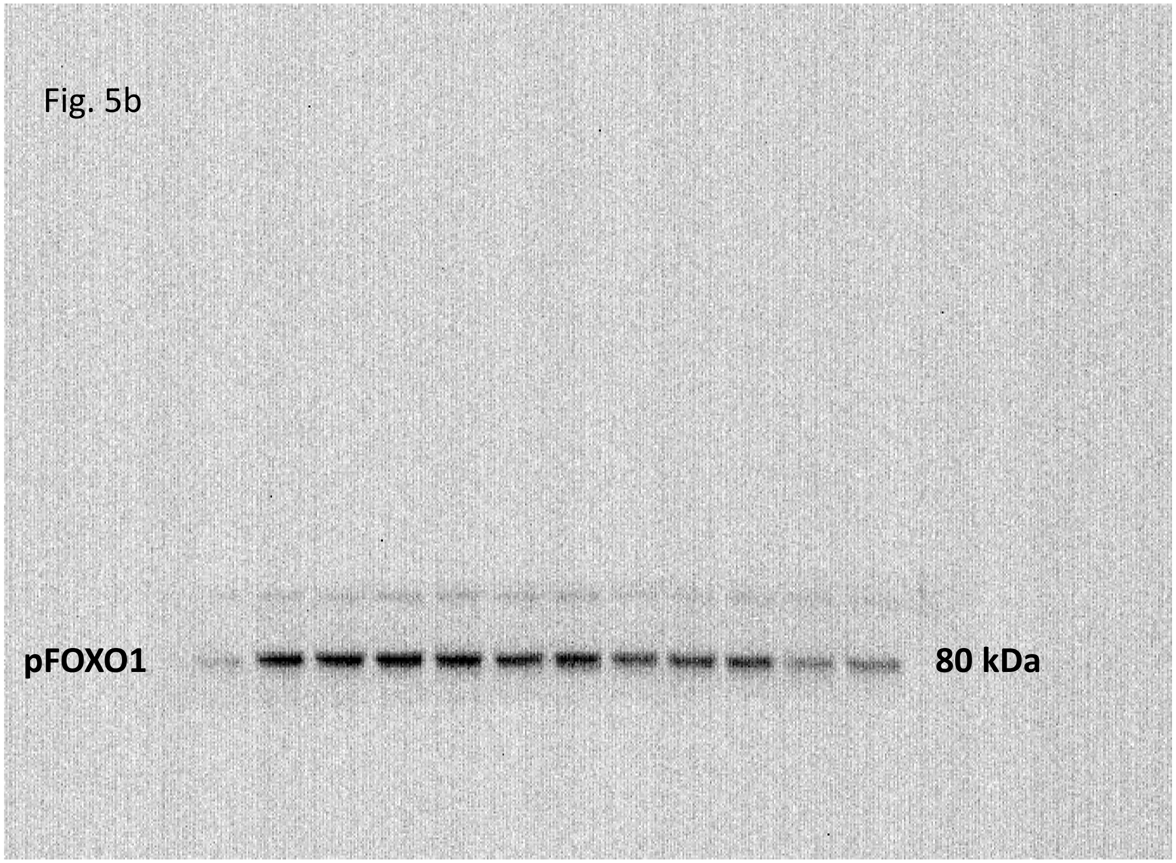

Fig. 5b

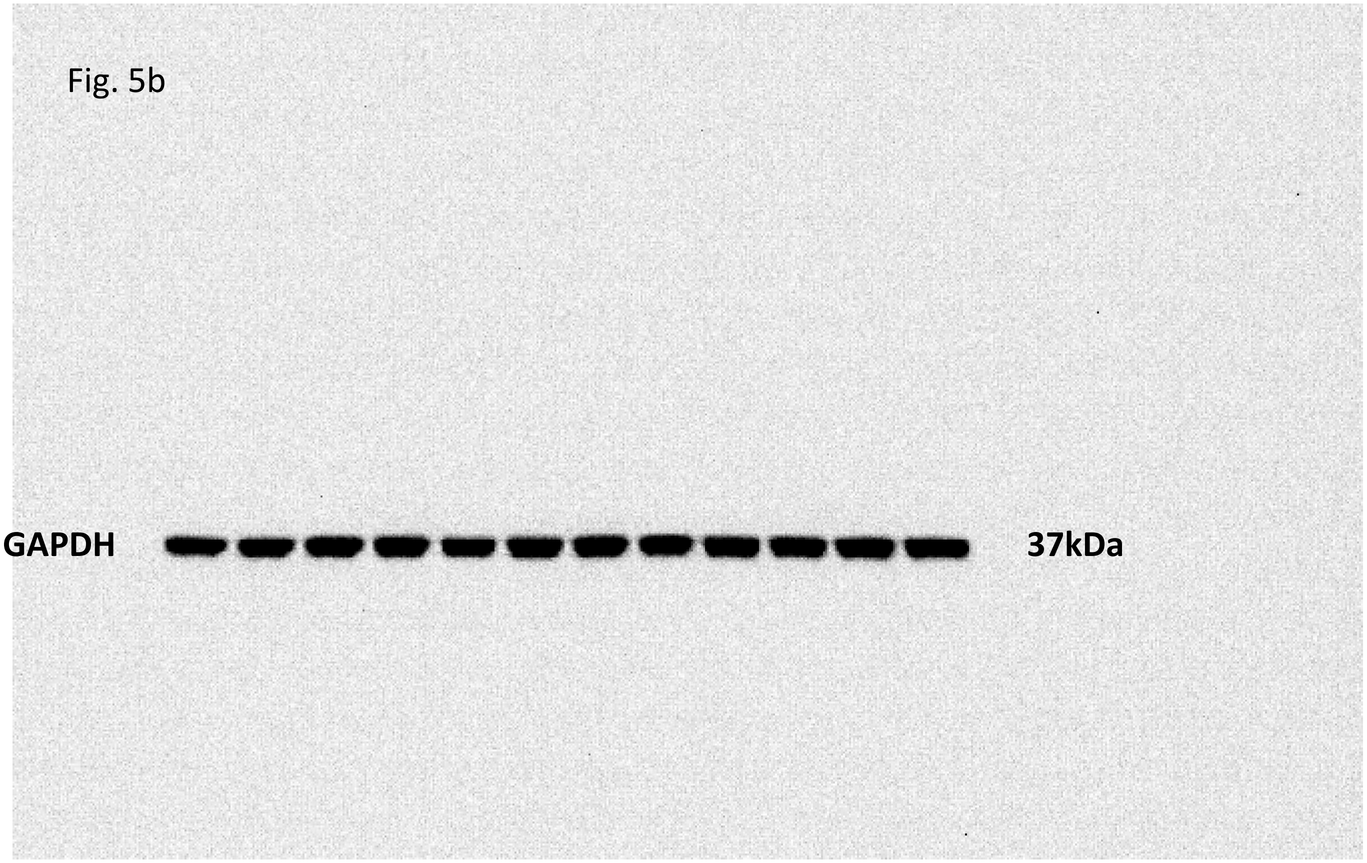

Fig. 5b

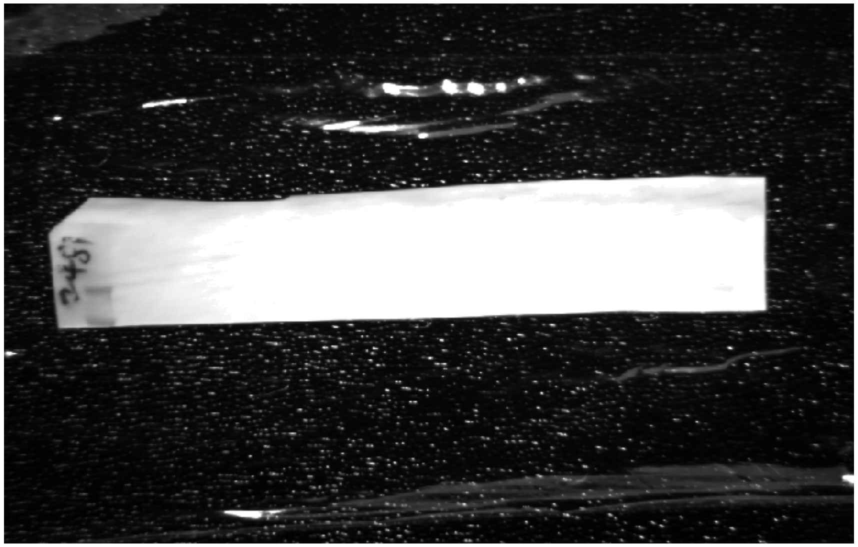

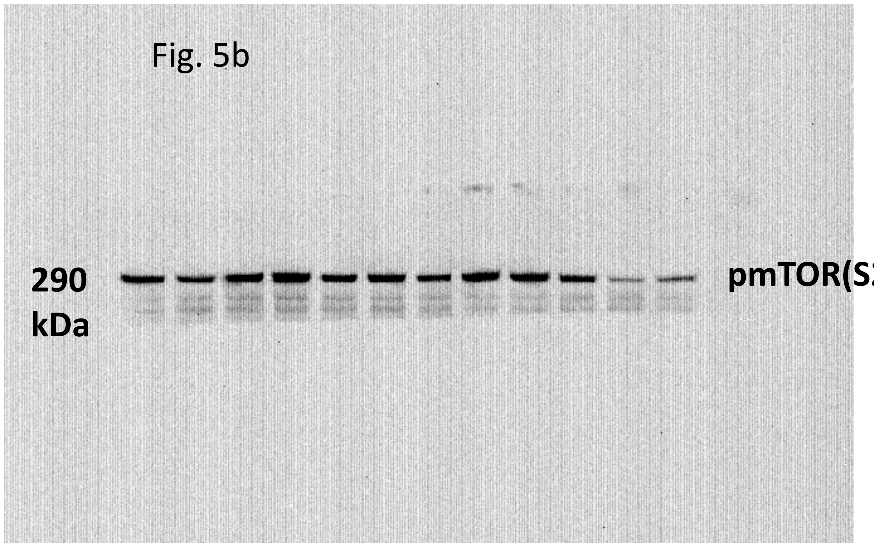

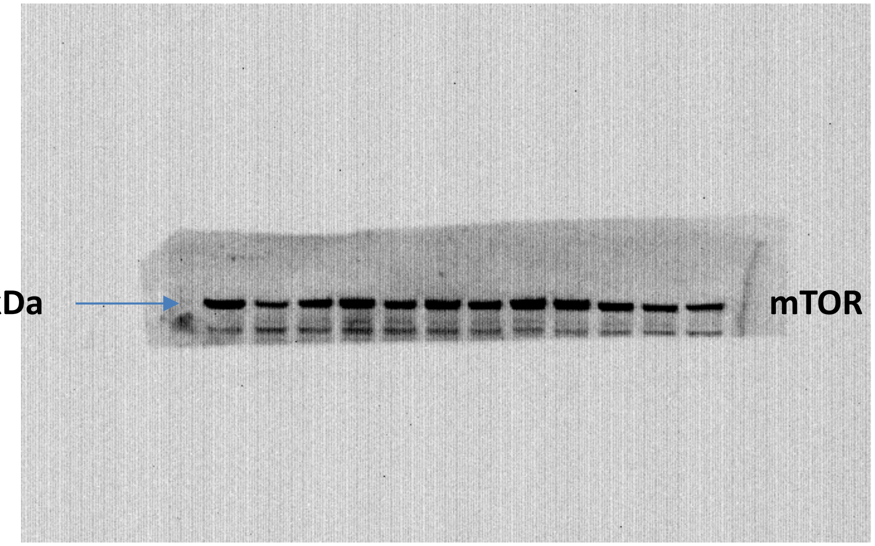

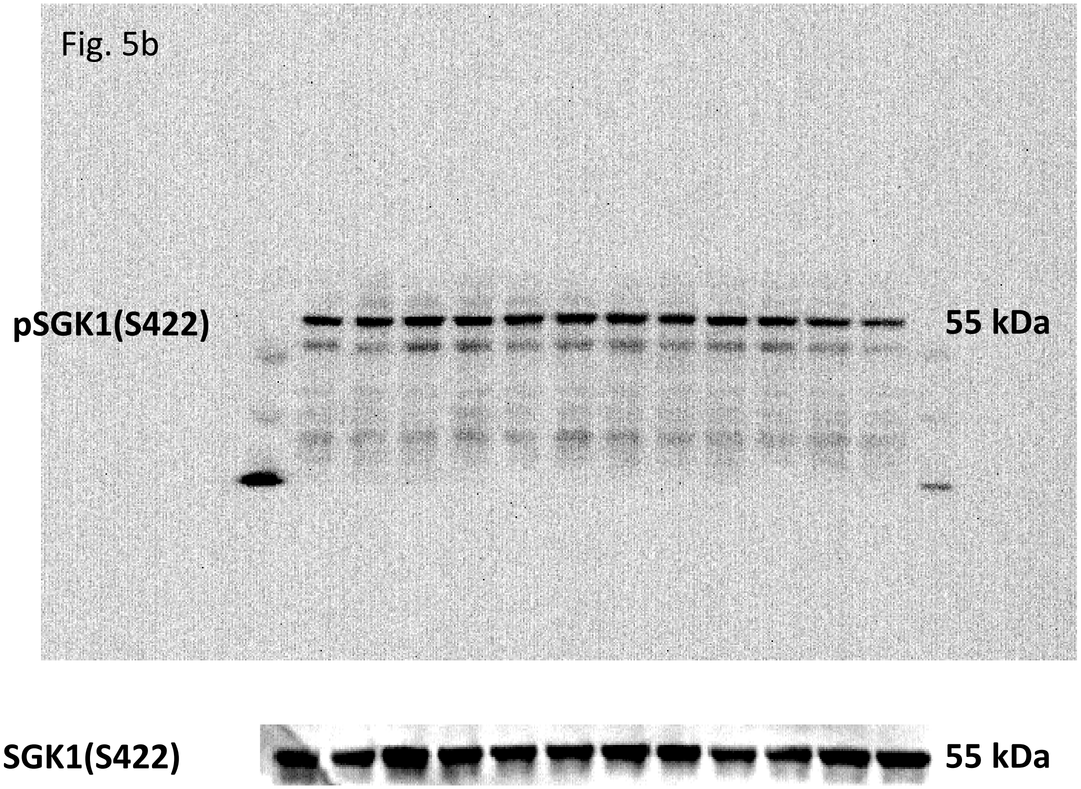
Fig. 5b
pSGK1(S422)
55 kDa
SGK1(S422)
55 kDa

Fig. 5c

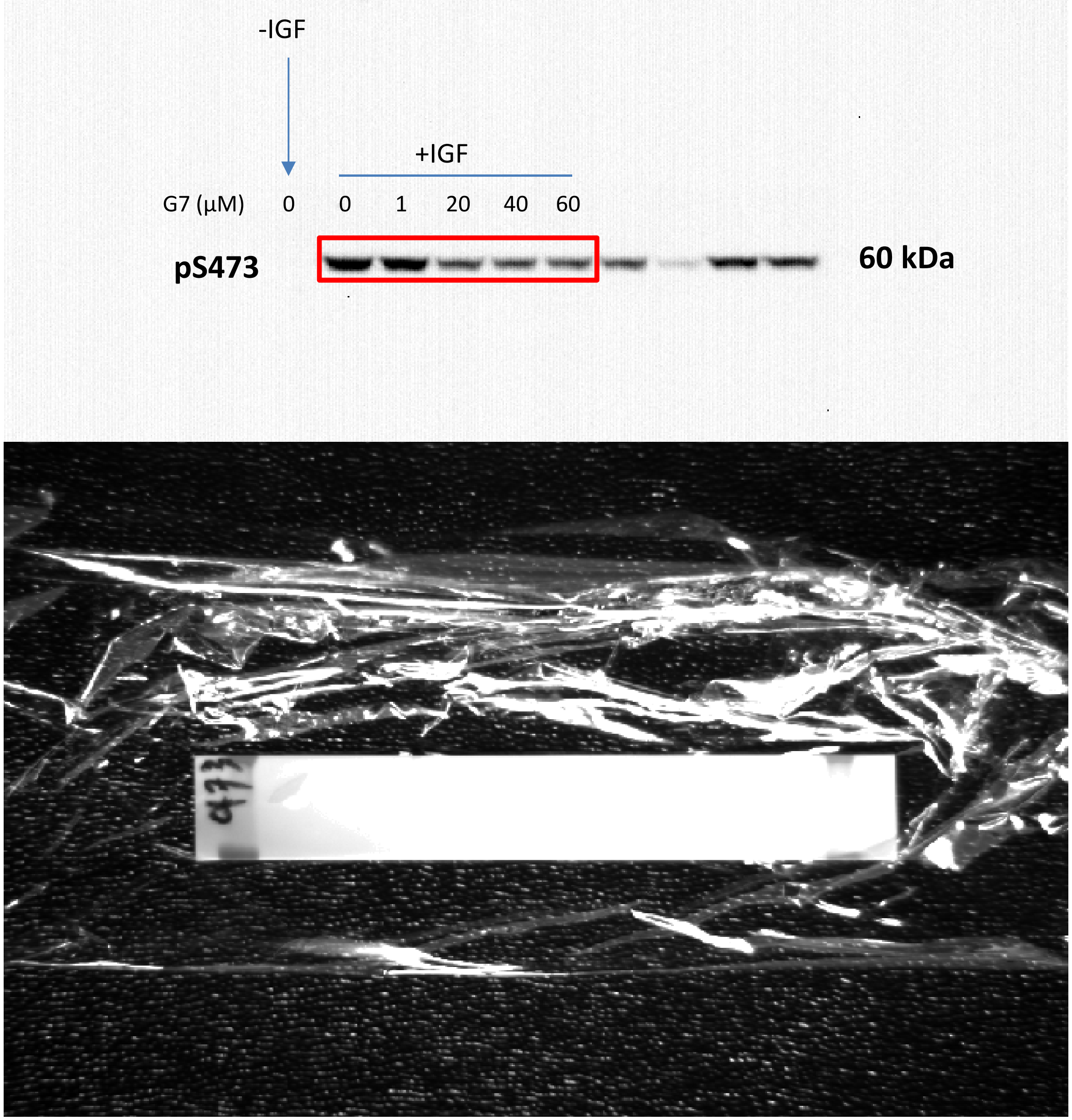

Fig. 5c

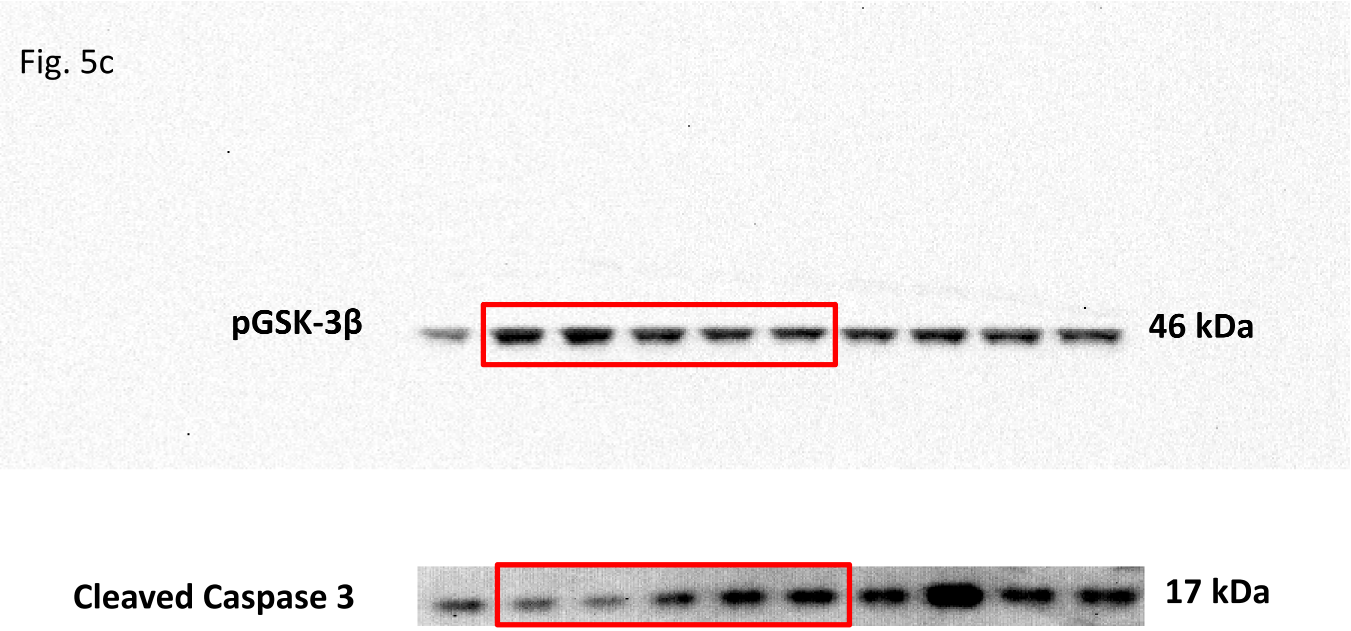

Fig.5c

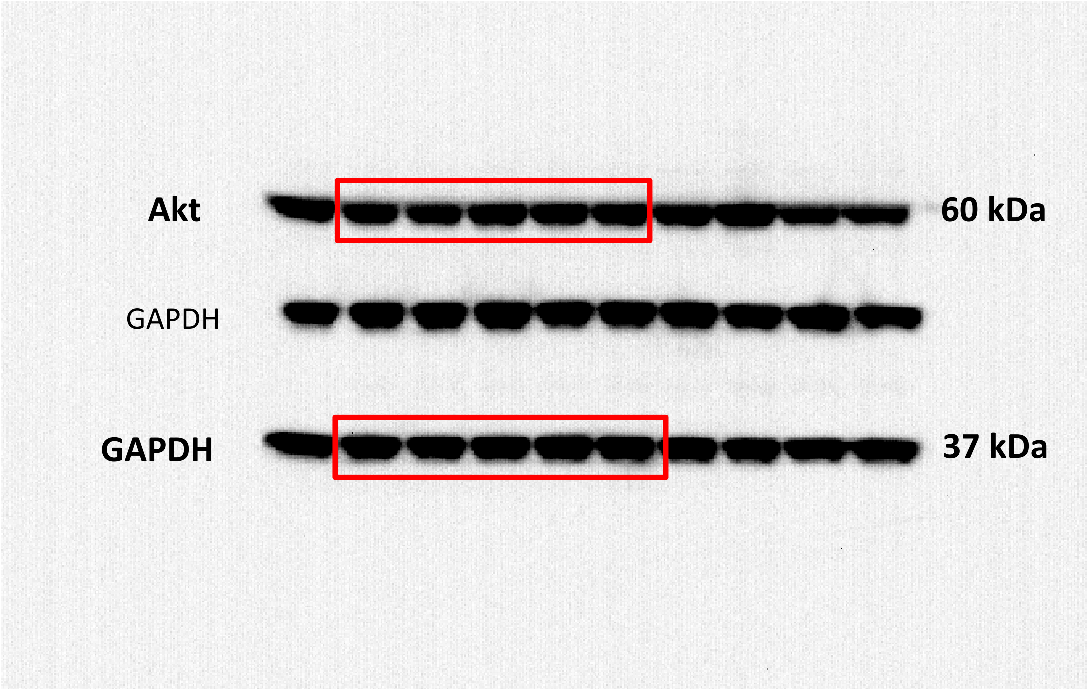
Akt
60 kDa
GAPDH
GAPDH
37 kDa

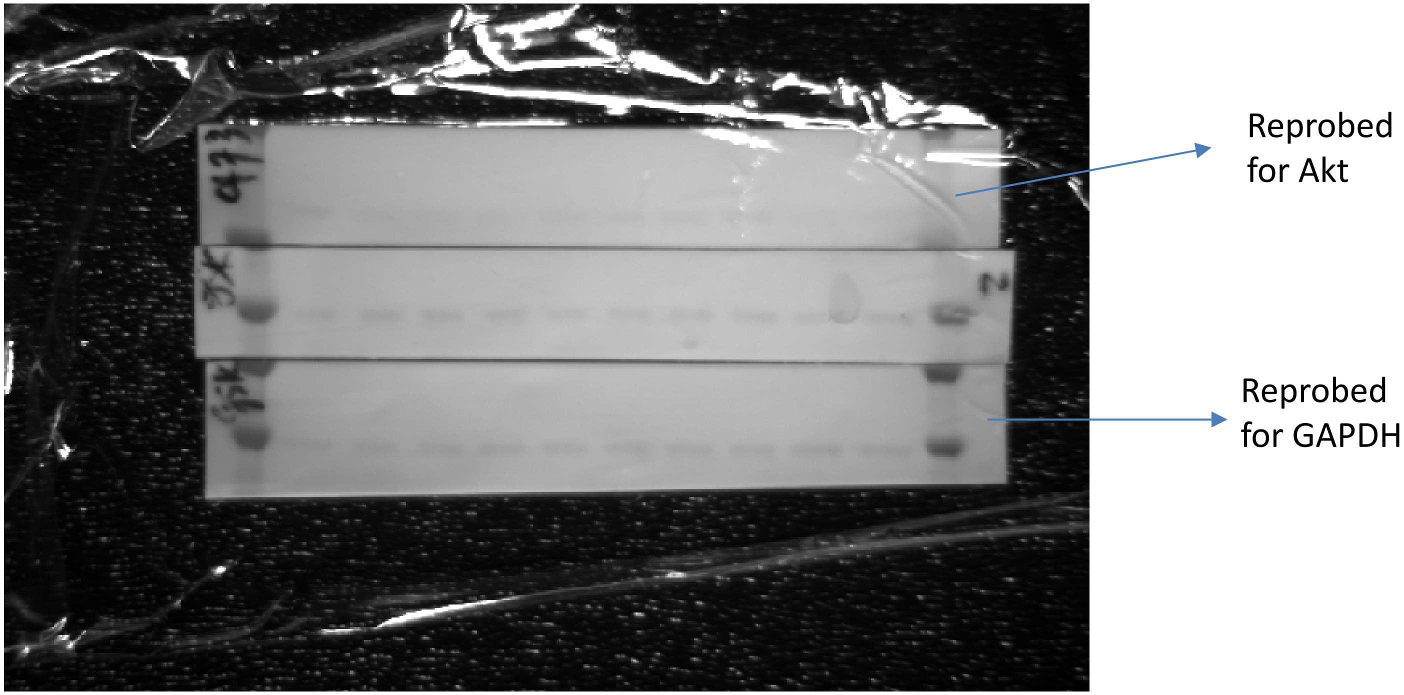
Reprobed for Akt
Reprobed for GAPDH

Fig. 5d

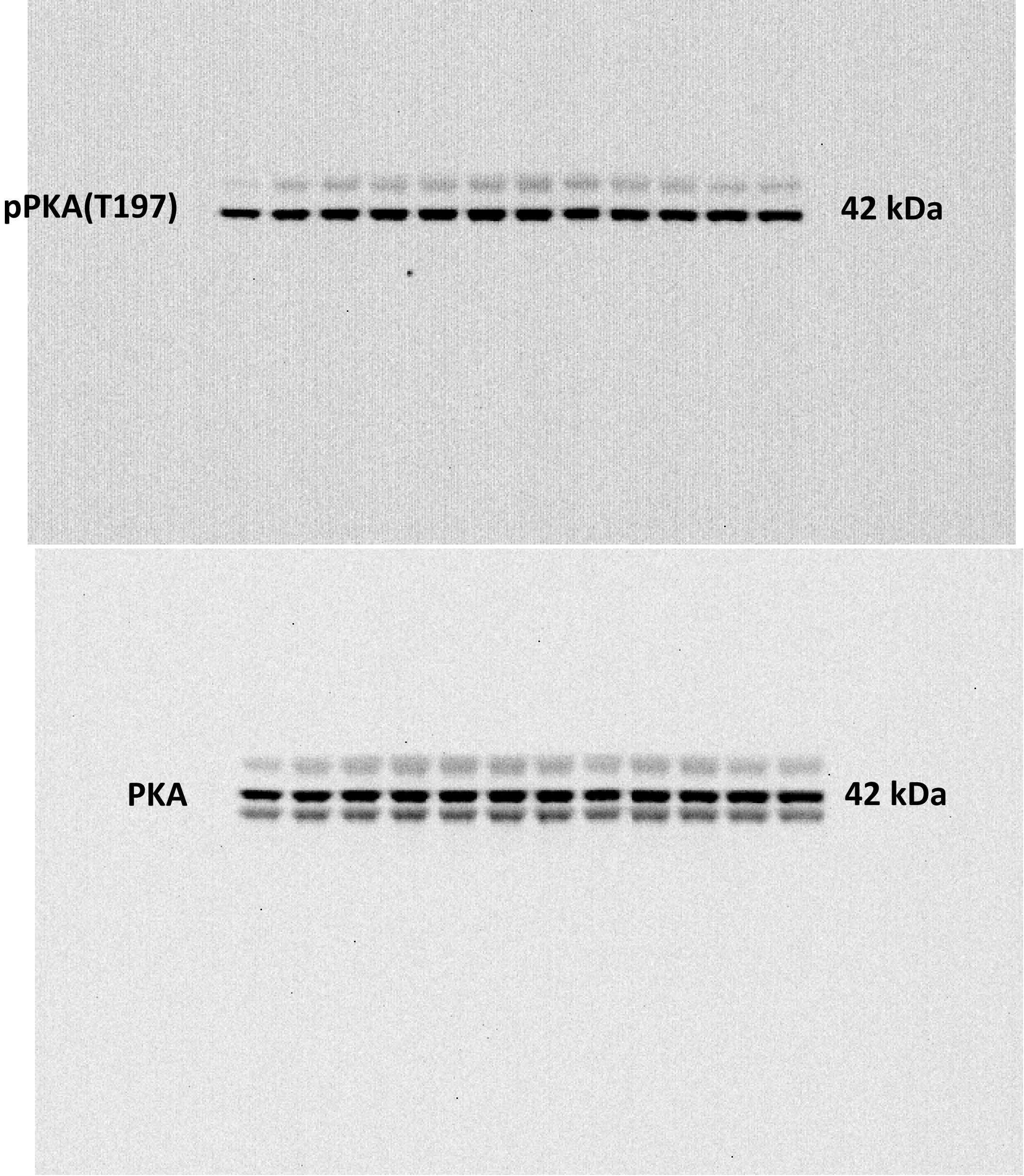

Fig. 5d

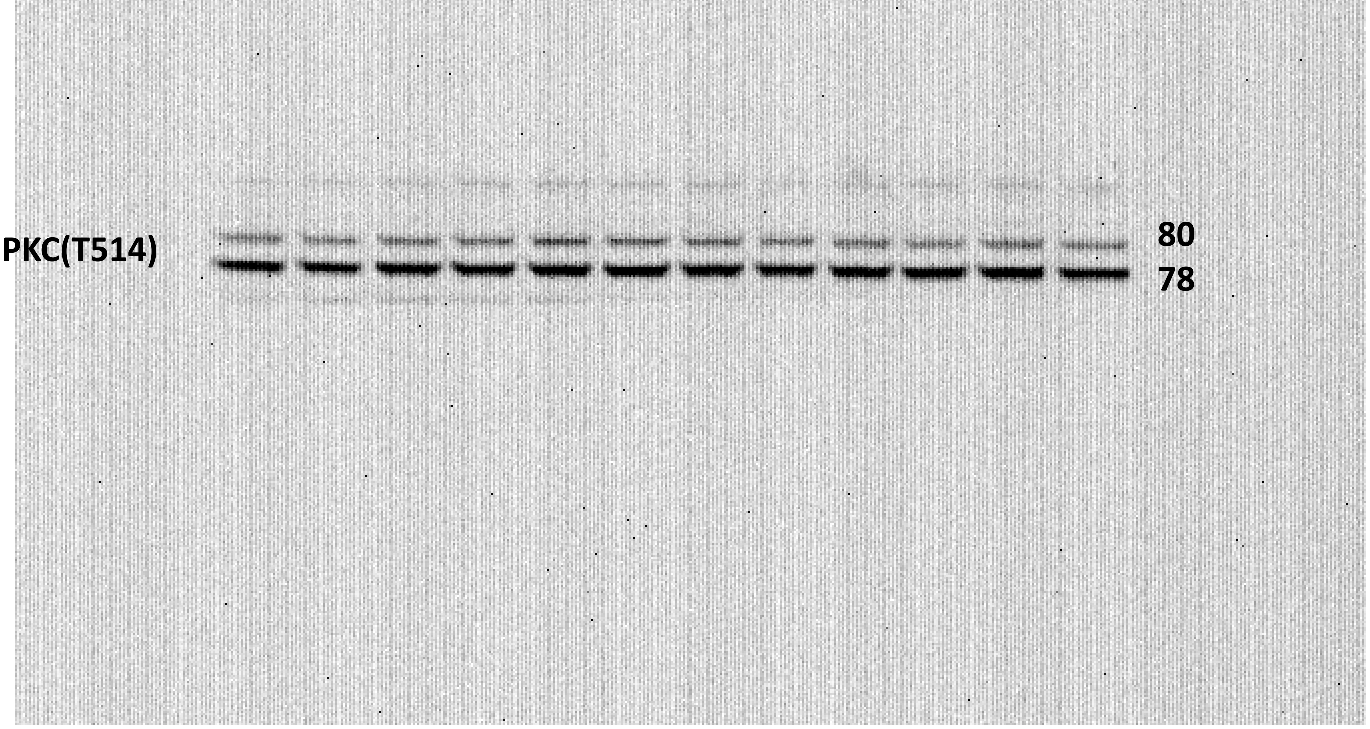

Fig. 5d

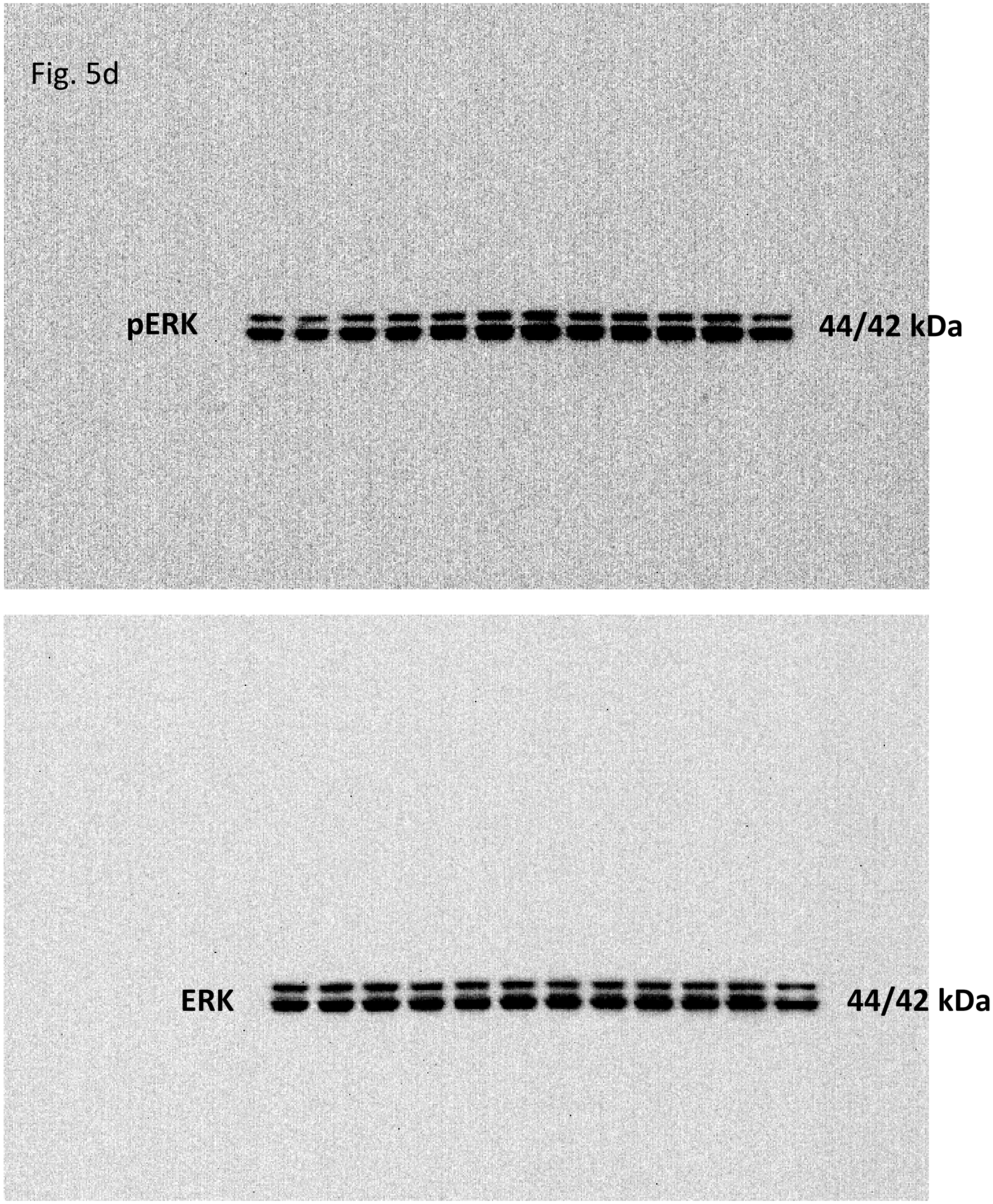

Fig. 5d

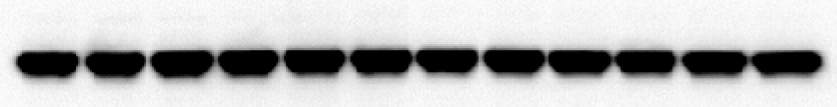

Fig. 7a left

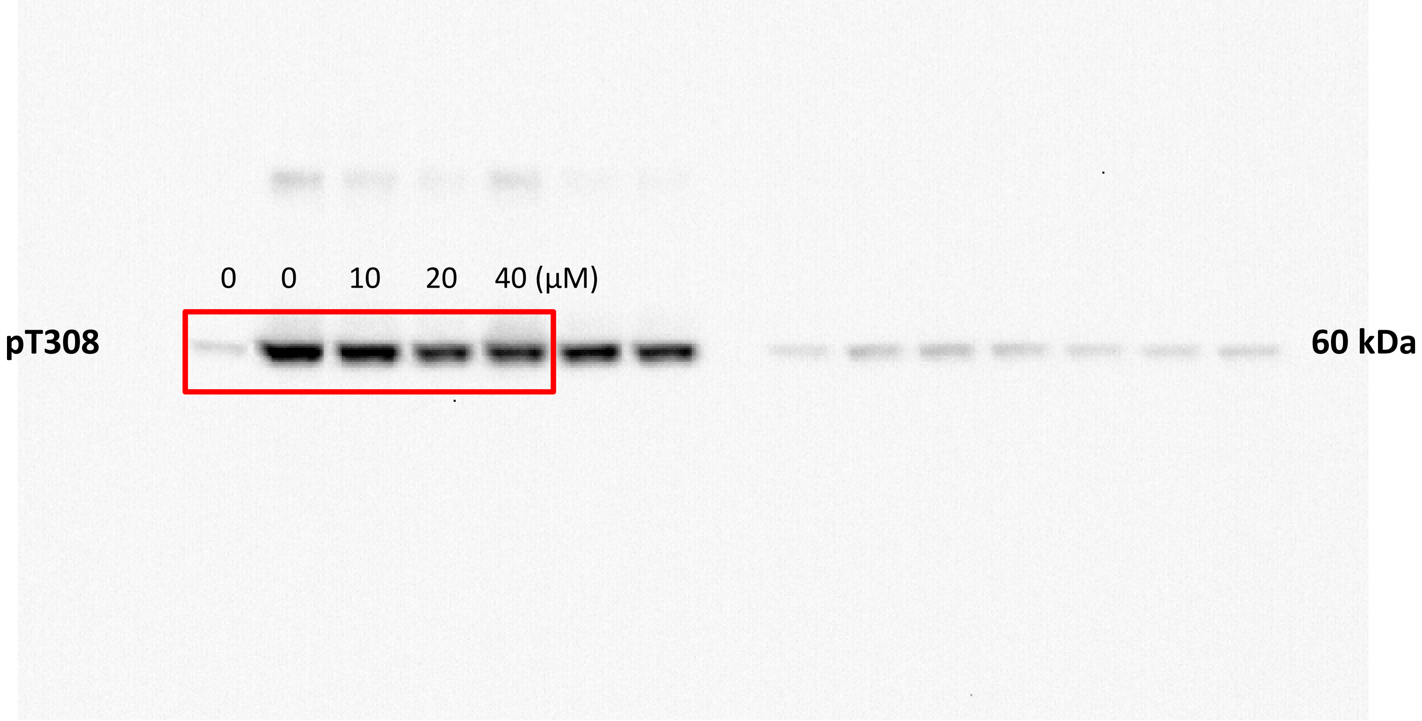

Fig. 7a right

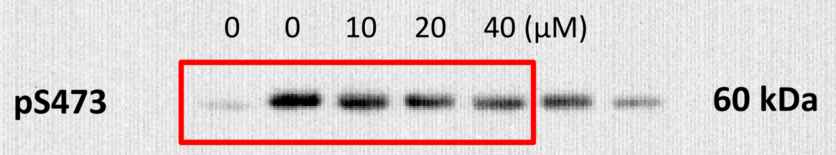

Fig. 7a

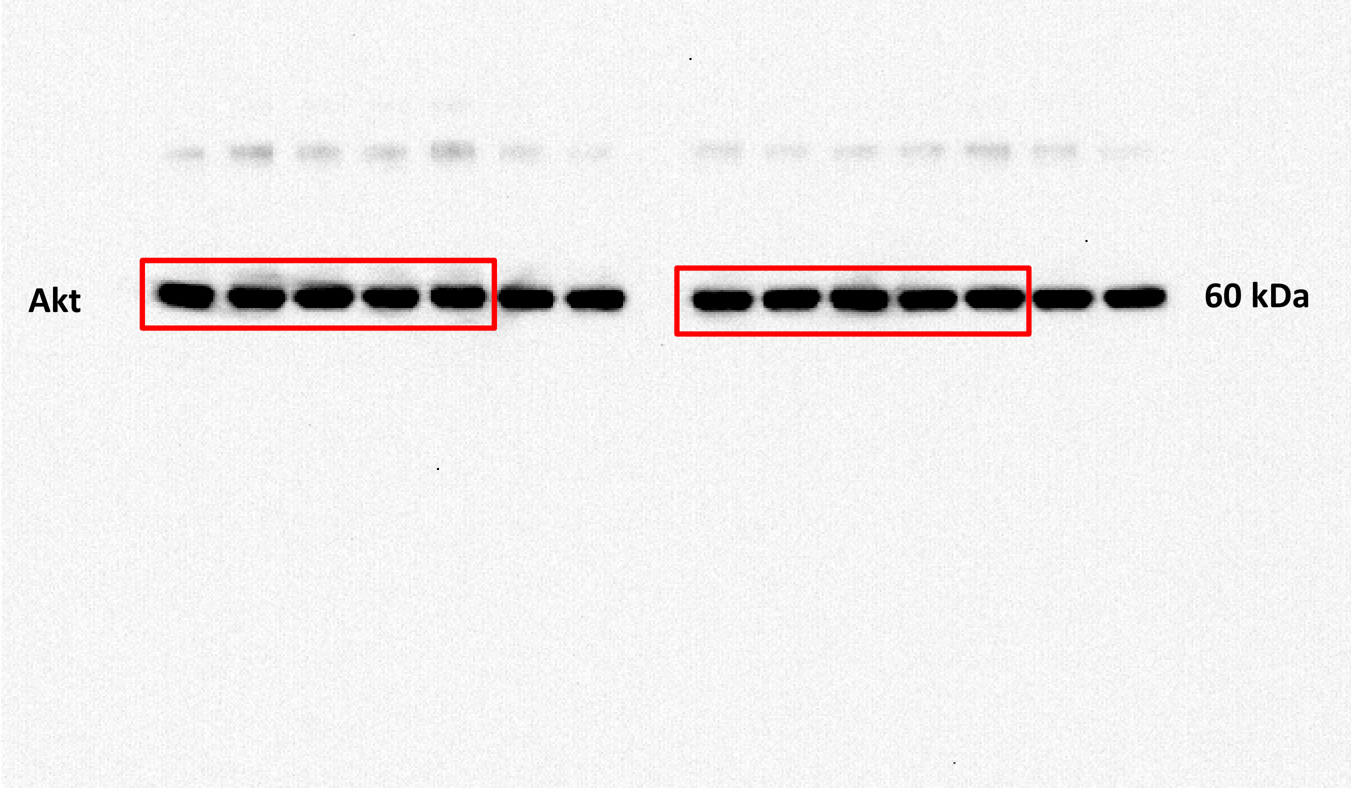

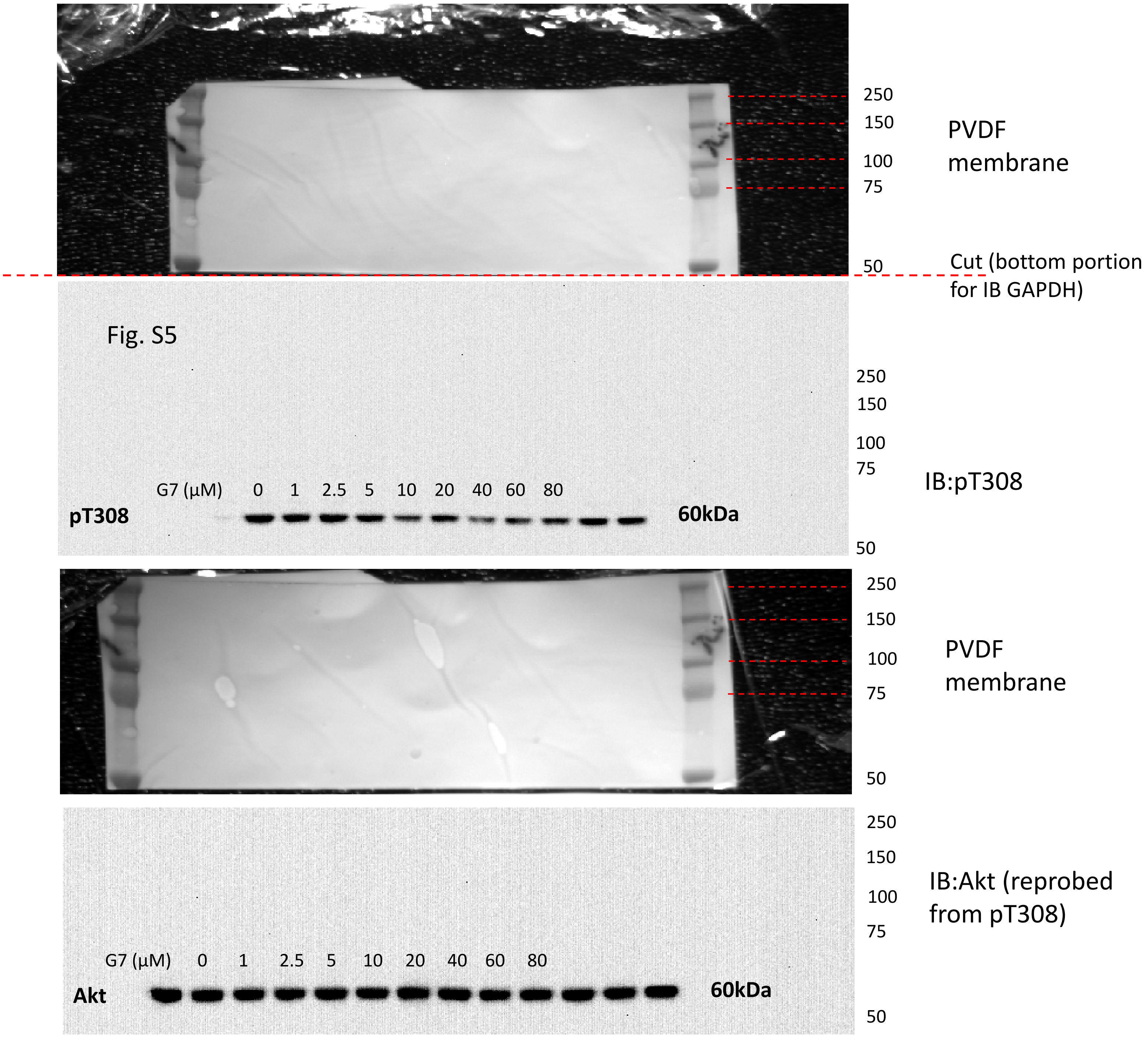

250
150
100
75
50
PVDF membrane
Cut (bottom portion for IB GAPDH)
Fig. S5
250
150
100
75
50
IB:pT308
G7 (μM) 0 1 2.5 5 10 20 40 60 80
pT308
60kDa
250
150
100
75
50
PVDF membrane
250
150
100
75
50
IB:Akt (reprobed from pT308)
G7 (μM) 0 1 2.5 5 10 20 40 60 80
Akt
60kDa

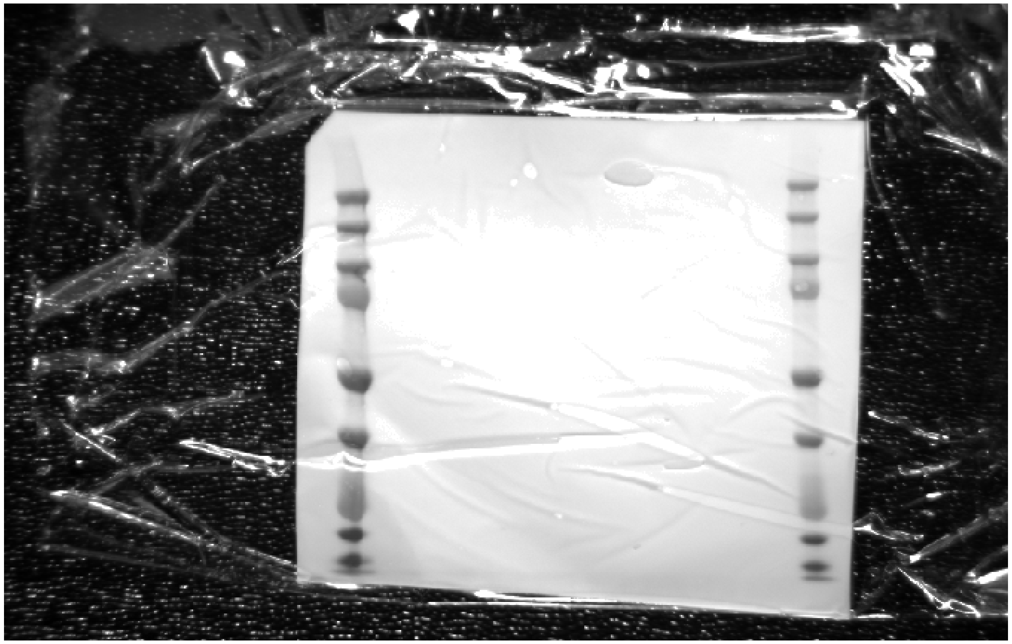

Fig. S7

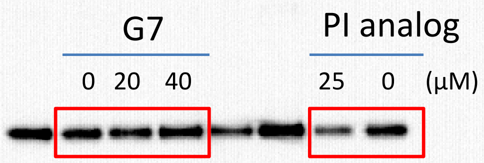

Supplement: Supplementary file 1 — Supporting information [file 41598_2017_11870_MOESM1_ESM.pdf]
